# Supplementary material for: TAS3 miR390-dependent loci in non-vascular land plants: towards a comprehensive reconstruction of the gene evolutionary history
Source: PeerJ. 2018 Apr 16;6:e4636. doi: 10.7717/peerj.4636 (PMC5907777; doi:10.7717/peerj.4636)
Supplement: Figure S1 [file peerj-06-4636-s001.doc]

**Figure S1.**

**Nucleotide sequences of TAS3 loci in mosses of *Sphagnum* and *Takakia* genera.**

Nusleotide sequences complementary to miR390 are in yellow.

***Sphagnum angustifolium*** Sphan-285 **(accession MF682529)**

GGCGGTAACCCTTCTGAGCTAAGTTTAAACGGATAGGGTTTGTGTTTTGCAAGTAGATTTGTGTGTTTTTTAATGTCTTTTAGTAAGGAAGGAAGCTGAATGTTAGGGTTAACATAATTATTATGTTTTTAGTATAAGCCCTTGTTTCAGATATGAATTCTATAGCTTGAAGACATGACAAACATGTTGTTCGTCATCTCATGATCACCTGCAGACCTACCCTTGAGACAAAATGTTTGCACATTATTGCAACATCTTGTCAATTTAGTTATCACTCCTGAGCTA

***S. girgensohnii*** Sphgi-292 (**accession MF682530)**

GGCGGTAACCCTTCTGAGCGTAAGTTTAAGCAAGATAGGGTTTGTGTTTTGCAAGTAGATTTGTGTATGTGTTTTTTAATGTCTTTCAGAAAGGAAGGAAGCTGAATGTTAGGGTTAACATAATTATTTATGTTTTTAGTATAAGCCCTTGTTTCAGATTTGAATTCTATAGCTTGAAGACATGACAAACATGTTGTTCGTCATCTCATGATCACCTGCAGACCTACCCTTGAGACAAAGTGTTTGCACATTATTGCAACATCTTGTCAATTTAGTTATCACTCCTGAGCTA

***S. fallax*** contig super_37 (**accession SRX2120232)**

GGCGGTAACCCTTCTGAGCTAAGTTTAAACGGATAGGGTTTGTGTTTTGCAAGTAGATTTGTGTGTTTTTTAATGTCTTTTAGTAAGGAAGGAAGCTGAATGTTAGGGTTAACATAATTATTATGTTTTTAGTATAAGCCCTTGTTTCAGATATGAATTCTATAGCTTGAAGACATGACAAACATGTTGTTCGTCATCTCATGATCACCTGCAGACCTACCCTTGAGACAAAATGTTTGCACATTATTGCAACATCTTGTCAATTTAGTTATCACTCCTGAGCTA

***S. fallax*** Sphfalx0293s0011 (**accession Sphfalx0293s0011)**

GGCGGTAACCCTTCTGAGCTAAGTTTAAACGGATAGGGTTTGTGTTTTGCAAGTAGATTTGTGTGTTTTTTAATGTCTTTTAGTAAGGAAGGAAGCTGAATGTTAGGGTTAACATAATTATTATGTTTTTAGTATAAGCCCTTGTTTCAGATATGAATTCTATAGCTTGAAGACATGACAAACATGTTGTTCGTCATCTCATGATCACCTGCAGACCTACCCTTGAGACAAAATGTTTGCACATTATTGCAACATCTTGTCAATTTAGTTATCACTCCTGAGCTA

***S. recurvum*** Sphre-283 (**accession SRX1513231)**

GGCGGTAACCCTTCTGAGCTAAGTTTAAGCGGATAGGGTTTGTGTTTTGCAAGTAGATTTGTGTTTTTTAATGTCTTTTAGTAAGGAAAGAAGCTGAATGTTAGGGTTAACATAATTATTATGTTTTTAGTATAAACCCTTGTTTCAGATATGAATTCTATAGCTTGAAGACATGACAAACATGTTGTTCGTCATCTCATGATCACCTGCAGACCTACCCTTGAGACAAAGTGTTTGCACATTATTGCAACATCTTGTCAATTTAGTTATCACTCCTGAGCTA

***S. recurvum*** Sphre-277 (**accession SRX1513231)**

GGCGATATCCTTTCTGAGCTAATTTGCGGATTTAAGGTTTGATTTGCAACATTAATGTGATGTTTTTGAGTTAGTATGGGTTATGAATTTAGTGATTCTTTTGTTTTTAAATCAACAATTATTGATCATTGCAAGAACAATAATGTTGATCGAATTCAAAAGTCATTTACTATGTTTGTACCCATCACCTCATTCTCATCTTGCAGACCTACCCTTGCGACAAAGTGTGTGCAGATTATTGCAACACCTTGTCAATTTCGATATCACTCCTGAGCTA

***S. magellanicum*** Sphma-285 (**accession SRX2330962)**

GGCGGTAACCCTTCTGAGCTAAGTTTGAGCGGATAGGGTTTGTGTTTTGCAAGTAGATTTGTGTGTTTTTTAATGTCTTTTAGTAAGGAAGGAAGCTGAATGTTAGGGTTAACATAATTATTATGTTTTTAGTATAAGCCCTTGTTTCAGATATGAATTCTATAGCTTGAAGACATGATAAACATGTTATTCCTCATTTCATGATCACCTGCAGACCTACCCTTGAGACAAAATGTTTGCTCATTATTGCAACATCTTGTCAATTTAGTTATCACTCCTGAGCTA

***S. magellanicum*** Sphma-286 (**accession SRX2330962)**

GGCGGTAACCCTTCTGAGCTaaattTtgagggaATAGGGTTTGAGCCTTGCAAGTAGAATTGTGAAATTTTTAATGTATTTCAGTAAGGAAGGAACATGAAAGTTACGGTTATCATAATTATTATGTTTTTAGTAAATGCCCTTGTTTGAAATATGACTTATATAGCTTGAAGACATAATAAAAAAGAAATTCATCATTTCATGACCTCCTGCACAACCTCCTTCGAGATAAAATGTTTGCACATTATTGAAACATCTCGTCAATTTAGTTATCACTCCTGAGCTA

***S. palustre*** Sphpa (**accession SRX1516347)**

GATTTGTGTGTTTTTTAATGTCTTTTAGTAAGGAAGGAAACTGAATGTTAGGGTTAACATAATTATTATGTTTTAGTATAAGCCCTTGTTTCAGATATGAATTCTATAGCTTGAAGACATGATAAACATGTTATTCCTCATCTCATGATCACCTGCAGACCTACCCTTGAGACAAAATGTTTGCACATTATTGCAACATCTTGTCAATTTAGTTATCACTCCTGAGCTA

***S. cribrosum*** Sphcri (**accession** [**ERX443237**](https://www.ncbi.nlm.nih.gov/sra/ERX443237%5Baccn%5D)**)**

GGCGGTAACCCTTCTGAGCTAAGTTTAAACAAGATAGGGTTTTGTGTTTTGCAAGTAGATTTGTGTGTGTGTTTTTAATGTCTTTCAGAAAGGAAGGAAGCTGAATGTTAGGGTTAACATAATTATTATGTTTTGAGAGATAAACCCTTGTTTCAGATTTGAATTCTATAGCTTGAAGACATGACAAACATGTTGTTCCTCATCTCATGATCACCTGCAGACCTACCCTTGAGACAAAATGTTTGCACATTATTGCAACATCTTGTCAATTTAGTTATCACTCCTGAGCTA

***S. lescurii*** Sphle (**accession ERX337183)**

GGCGATATCCTTTCTGAGCTAATTTGCAGATTAAGGTTTGCAACATGCATGTAATGTTTTTGAGTTAGTATGGGTTATGATTTTAGTGATTCTTTTGTTTCTAAATCAACAATTATTGATCATTGC

[***Takakia lepidozioides***](https://www.ncbi.nlm.nih.gov/Taxonomy/Browser/wwwtax.cgi?mode=Info&id=37425)Takle-207 (**accession ERX2100030)**

GGCGCTAACCTTCCTGAGCTAAGCCAGTAGAGGGTGGGTTGAGGGGGGGCACTAGGACACTTCCCGGCCTTGTGCCGGATATGGTGGCCTAGGGTGTGATGAGTGCTTTACCAGCACCTCACATTGGCCCAGCCGTCCTACCCTTGGTACAAGGGGACTGCAACTCTTTGCGCCATCCTTGTAAATTTGTTTATCACTCCTGAGCTA
