# Supplementary material for: TAS3 miR390-dependent loci in non-vascular land plants: towards a comprehensive reconstruction of the gene evolutionary history
Source: PeerJ. 2018 Apr 16;6:e4636. doi: 10.7717/peerj.4636 (PMC5907777; doi:10.7717/peerj.4636)
Supplement: Figure S2 [file peerj-06-4636-s002.doc]

**Figure S2.** **Putative ta-siRNA preceding tasiAP2 site in the same phase.**

**(A)** Nucleotide sequences and encoded amino acid sequences of moss mRNAs potentially recognized by the putative ta-siRNA preceding in the same phase tasiAP2 site of some moss TAS3 loci. TBLASTN was used at 1KP and NCBI blast sites. Complementary mRNA sequences are in violet; conserved amino acid sequence signatures are in yellow.

**(B)** Pairwise sequence comparisons of some predicted amino acid sequences encoded by plant transcriptoms with a protein encoded by moss ***Diphyscium foliosum* (AWOI_2068845)**. Conifers are in green; dicots are in blue; monocots are in brown.

**(A)**

**HVBQ_scaffold_2019036 *Tetraphis pellucida***

caagatgtcttacaacgacgacagcagccgaggcgataactacggtagcggcaatcagggc

K M S Y N D D S S R G D N Y G S G N Q G

aatactggcggcggcatgggacaagacgacagctatggctcctctggccggactggaggt

N T G G G M G Q D D S Y G S S G R T G G

gctcaaggcatggatgattccagctacggtactggggcccaaggtagcacgcgccagggt

A Q G M D D S S Y G T G A Q G S T R Q G

ggcgacgacagctacggatcctctggtcaaggcaacacgggcggtggtctcggaagagac

G D D S Y G S S G Q G N T G G G L G R D

gacacctatggctcatctggacagactggtggtgcccaggatgattcctacggctcatca

D T Y G S S G Q T G G A Q D D S Y G S S

ggaagaannnnnnnnnnnnnnnnnnnnnacacatatggctcgtctaaccagactggaggt

G R X X X X X X X X T Y G S S N Q T G G

gcccaacaaggaggctatggaggcgataattatagcagcggcggcaatcagcagactggt

A Q Q G G Y G G D N Y S S G G N Q Q T G

ggcggtctcggaactgatgatacctacggctctggaggcgctactggtggtgcatctagc

G G L G T D D T Y G S G G A T G G A S S

ggaatggaccaaggcgagtatggcgttggttcaggccgcagccagcagcaaaacctcggt

G M D Q G E Y G V G S G R S Q Q Q N L G

ggcgtaggtgccggcagcggaaactcgggaggcgataactacgataatgacaactcggga

G V G A G S G N S G G D N Y D N D N S G

agcaagaaaa

S K K

**AWOI_2068845 *Diphyscium foliosum***

cccaaaatgtcttacaacgacgacagcagccgaggcggtcagtacggcagcagcaacacg

P K M S Y N D D S S R G G Q Y G S S N T

ggaggcggcatgggacaagatgatagctacggctcctctggtcggactggaggtgctcaa

G G G M G Q D D S Y G S S G R T G G A Q

ggcacggatgattcaagctatggcactggagcccagggcggcatgcgccagggcggtgat

G T D D S S Y G T G A Q G G M R Q G G D

gacagctatggatcctcccgtcaaggcaacacaggtggtggcctcggccaagacgacacc

D S Y G S S R Q G N T G G G L G Q D D T

tacggctcctctcgccagactggtggtgcccaggacgattcctacggctcatcgggaaga

Y G S S R Q T G G A Q D D S Y G S S G R

gctggtggcgcaactggcggtggcctaggcgctgatgacacatatggctcctctaaccaa

A G G A T G G G L G A D D T Y G S S N Q

agtggaggtgcccaacgaggaggctatggaggcgatgacactacaagcggcggcagcaag

S G G A Q R G G Y G G D D T T S G G S K

cgtactgggggcggccttggagctgatgacaactacggcaacgaaggtgctactggcggt

R T G G G L G A D D N Y G N E G A T G G

gcttccagcggaatggaccagggcgagtacggcgtcggctctggccgtagccagcagcag

A S S G M D Q G E Y G V G S G R S Q Q Q

aacctcggaggtgttggtgcaggcagtggacagtcaggcggcgatacctacgatgacggc

N L G G V G A G S G Q S G G D T Y D D G

aattcgggtggcaagaaggactctacaatgggcaagcttatggaaaaggctggtggcatg

N S G G K K D S T M G K L M E K A G G M

ttgaaaaatgagaagcttgaacagaagggcgcgcagaagcgagctgaacagggtgggtac

L K N E K L E Q K G A Q K R A E Q G G Y

gatgacaccaccggtgggagcggtggctatggcggcgacaacactagctccggctatggt

D D T T G G S G G Y G G D N T S S G Y G

gacaacactggcagcggtcgcggcggtgacaactactgagccggccagcgcggcagaagt

D N T G S G R G G D N Y - A G Q R G R S

tgagatgggtggataagcgatttgagctaaaatgtatacctctacgacggc

- D G W I S D L S - N V Y L Y D G

**FFPD_2011091 *Ceratodon purpureus***

gaactctaacagcactaaacactccaaatacatccaactgttcccttcaatatgtcttac

E L - Q H - T L Q I H P T V P F N M S Y

aacgacgacagcagccgaggtgataactacggcagcagcaacacaggaggcggccgagga

N D D S S R G D N Y G S S N T G G G R G

caagacgatagctatggctcatctggtcgaaccggaggcggtctaggacaagacgatagc

Q D D S Y G S S G R T G G G L G Q D D S

tacggctcatctgaccgaaccggaggtgctcaaggcttggatgattccagctacggcact

Y G S S D R T G G A Q G L D D S S Y G T

ggcacccagggcggcctgcgccagggcggtgatgacagctacggatcctctcgtcagggt

G T Q G G L R Q G G D D S Y G S S R Q G

aacactggtggtggccttggacaagacgacacttacggctcctctcgtcagactggtggt

N T G G G L G Q D D T Y G S S R Q T G G

gcccaggacgattcctatggctcatcaggaagaactggcggaacaactggcggtggtcta

A Q D D S Y G S S G R T G G T T G G G L

ggtgccgatgacacatatggctcgtctaaacaaaccggaggtgctcaacaaggaggctat

G A D D T Y G S S K Q T G G A Q Q G G Y

ggaggcgacgactattccagtgggggtaacaagcgtactggtggcggtcttggagctgat

G G D D Y S S G G N K R T G G G L G A D

gacacttacggcagcgaaggtgctactggtggtgcctctagcggancagggcgaatatgg

D T Y G S E G A T G G A S S G X G R I W

tgtcggctctggccgcagccagcagcagaatcttgggggtgttggtgcaggaggcgggag

C R L W P Q P A A E S W G C W C R R R E

gtcgaatgacgatagctacgatgatggcagctcgggtggcaagaaggattccacaatggg

V E - R - L R - W Q L G W Q E G F H N G

caagctgatggagaaggctggtggcatgttgaagaatgagaatcttcaacagaagggcgc

Q A D G E G W W H V E E - E S S T E G R

ccagaagcgagccgagcagggagcatatgatgacactactggtggtagcggtgggtacgg

P E A S R A G S I - - H Y W W - R W V R

cggtgacaataccagctctggctatggagacaacagtggccgcagtggtggctatggtga

R - Q Y Q L W L W R Q Q W P Q W W L W -

cgacaacacgagctccggctatggaggcaatactgggagtggtcgtggtggagacaatta

R Q H E L R L W R Q Y W E W S W W R Q L

ctgagctgccagcgcagcaccagttgagataggtgcacaaagccatatggg

L S C Q R S T S - D R C T K P Y G

**RDOO_2091453 *Racomitrium varium***

aacaacttgtctccaaagaacgtgaacagatatcgactaaacccacacccacccaaacata

T T C L Q R T - T D I D - T H T H P N I

cccaccaagatgtcttacaacgacgacagcagccgaggcgacaactatggtagtggcaac

P T K M S Y N D D S S R G D N Y G S G N

actggaagtggcatgggacaagatgacagctacggctcctctggtcggactggaggcggt

T G S G M G Q D D S Y G S S G R T G G G

atgggacaagacgatagctttggctcctctggtcggactggaggtgctcaaggtatgg

M G Q D D S F G S S G R T G G A Q G M

**VMXJ_2127900 *Leucobryum albidum***

cctaacttgactccgttcggcctctcttctgtcttctatttaaacccgtcattctccgcgcc

- L D S V R P L F C L L F K P V I L R A

acactttacttcatcgaacaaagaacttactcaattacctgtactagcaagcttcaaacc

T L Y F I E Q R T Y S I T C T S K L Q T

tttcaaatccttctctcaccacacttttcttaaacagcaatcatgtcttacaacgacgat

F Q I L L S P H F S - T A I M S Y N D D

caagacagttacggatcatctggacgtaccggaggtggtctagggcaagatgatagctac

Q D S Y G S S G R T G G G L G Q D D S Y

ggctcatctaaccaggctggaggcaacagtggactcgggcagcaggatgattcctacggg

G S S N Q A G G N S G L G Q Q D D S Y G

tcatctggtcgcactggaggtggcctcggccaagacgacagctatggttcctctggccgc

S S G R T G G G L G Q D D S Y G S S G R

accggtggtaacagtggacttggacagcaggatgactcatatggatcatctggccgcact

T G G N S G L G Q Q D D S Y G S S G R T

ggtggcggcctcggtaaagatgatagctacggctcctcaggacgtaccgggggaaataca

G G G L G K D D S Y G S S G R T G G N T

ggtttcgggcaggacgacagctacggagatacagcaacttctggcaggactggtggtggc

G F G Q D D S Y G D T A T S G R T G G G

cttggacaagatgattcgtatggatcctctggacaaactggcggtaacagcggatacggc

L G Q D D S Y G S S G Q T G G N S G Y G

caaggcgatagttatggagactcaactacctcaggtcggactgccggcggcctaggacaa

Q G D S Y G D S T T S G R T A G G L G Q

gatgattcctacggcagcaacactcaaactggcggcgcccagggcacaggccagggcgac

D D S Y G S N T Q T G G A Q G T G Q G D

aactatggtctgggaagtacccgcactcaggctcagcagcagggaggcctcggcggtagt

N Y G L G S T R T Q A Q Q Q G G L G G S

ggtgggaacgatgactatgacgacaactccggcagcaagtcatccaagaagcaagactct

G G N D D Y D D N S G S K S S K K Q D S

accgctggcaagcttatggaaaaagctggcggcttgttcaagaacgaaaaattagctcaa

T A G K L M E K A G G L F K N E K L A Q

aagggacaggaaaagagagatgctgctggtgcatatgatgataccacaggtggcagtgga

K G Q E K R D A A G A Y D D T T G G S G

tacggtaatactcaaagtggtggcggatacgacgataatcaaagtgggggtggatacgac

Y G N T Q S G G G Y D D N Q S G G G Y D

aaccaaagcggcggccgagacaactactaaagttcttattgcctcgattcgagagcgtcg

N Q S G G R D N Y - S S Y C L D S R A S

ataacgatcaactgccgacaccaattgagattgaaaggaaaacaaaacaaagttgctggg

I T I N C R H Q L R L K G K Q N K V A G

ctgtattatcaaatcaattaagggctatgtatcttcgtgtacctgctatagcttctaagc

L Y Y Q I N - G L C I F V Y L L - L L S

atggttaacacccgaaaaaa

M V N T R K

**(B)**

***Picea glauca* (accession GCHX01156184)** (E-value: 9e-12)

Query 1 MS-YNDDSSRGG--QYG--SSNTGGGMGQDDSYG--SSGRTGGAQGTDDSSYGTGAQGGM 53

MS YND+ S G + G SSNT GG ++YG S R GG G DDS T G +

Sbjct 111 MSNYNDNDSYGSNERRGNDSSNTYGGNDSSNTYGGGDSNRVGGLGG-DDSYSSTKRSGDI 287

Query 54 -RQGGDDSYGSSRQGNTGGGLGQDDTYGSSRQTGGAQDDSYG--SSGR-------AGGAT 103

GG +S+G+S N GG+G DD+YGS++++G DD+YG S+ R GG+

Sbjct 288 DSYGGTESFGNS--ANPSGGIGADDSYGSTKRSG--NDDNYGNDSTSRNEYGSGTTGGSG 455

Query 104 GGGLGADDT-YGSSN-QSGGAQRGGY-GGDDTTSGGSKRTGGGLGADDNYGNEGATGGAS 160

G +DD+ YG SN +SG + Y GG+D S GS TGG G +GN+ ++GG

Sbjct 456 FGNKTSDDSSYGDSNTKSGRSDNNAYSGGND--SYGSGATGG-AG----FGNK-SSGGGD 611

Query 161 SGMDQGEYGVGSGRSQQQNLGGVGAGSGQSGGDTYDDGNSGG-----KKDSTMGKLMEKA 215

S D +G G GSG +GG Y GN G K DST GKL+EKA

Sbjct 612 STYDNSNTRLGKSDDTSAYSGSNEYGSGTTGGAGY--GNKSGDNDNKKNDSTAGKLLEKA 785

Query 216 GGMLKNEKLEQKGAQKRAEQG-GYDD---TTGGSGGYGGDNTSSGYGDNTGSGRGGDNY 270

GG+ KN+ L +KG KR E+G G DD +TG YGG + YG GS R ++Y

Sbjct 786 GGLFKNDGLAEKGRVKREEKGFGQDDSYGSTGNDNSYGGSTNDNSYG---GSERRNNDY 953

***Araucaria cunninghamii* (accession GCKF01033247)** (E-value: 5e-06)

Query 15 SSNTGGGMGQDDSYGSSGRTGGAQGTDDSSYGTGAQGGMRQGGDDSYGSSRQGNTGGGLG 74

S+ T GG G DDSYGSSGRT G TD S+G GAQ G TGG L

Sbjct 858 STRTTGGYG-DDSYGSSGRTAGLGDTD--SFG-GAQKKAAGYGGGDDSYGSGARTGG-LA 694

Query 75 QDDTYGSSRQTGGAQDDSYGSSGRAGGATGGGLGADDTYGSSNQSGGAQRGGYGGDDTTS 134

D YGS +T DDSYGS GR + G G D++YGS ++ G DT S

Sbjct 693 DSDNYGSGGRTARLGDDSYGSGGRTTTDSYGA-GGDNSYGSGGRTSGRG-------DTDS 538

Query 135 -GGSKRTGGGLGADDNYG--NEGATGGA---------SSGMDQGE-YGVGSGRSQQQNLG 181

G + RT G LG DD YG ++ T +S + + + YG + R+ G

Sbjct 537 YGANTRTAG-LG-DDTYGSAHKKTTEYGGESYGSGGRTSALTEDDSYG-SADRTTGGGYG 367

Query 182 GVGAGSGQSGG----DT-Y-DD-GNSGGKKDSTMGKLMEKAGGMLKNEKLEQKGAQKRAE 234

GS + GG D Y DD S DST GKLMEKAGGML ++KL+Q+G++KRA

Sbjct 366 DDSYGSSKKGGLGSDDVSYGDDQRRSDSTGDSTTGKLMEKAGGMLGSKKLQQQGSEKRA- 190

Query 235 QGGYDD 240

Q DD

Sbjct 189 QAREDD 172

***Abies pinsapo* (accession GCZN01069599)** (E-value: 1e-11)

Query 84 QTGGAQDDSYGSSGRAGGATGGGLGADDTYGSSNQSGGAQRGGYGGDDT-T--SGGSK-- 138

Q+G D+YGS + G T G L + DTYGS N S G YG +T T SG +

Sbjct 684 QSGLDSSDTYGSGNTSTGRTKG-LDSSDTYGSGNTS----SGNYGSSNTDTLGSGNTSSG 520

Query 139 RTG-GGLGADDNYGNEGATGGASSGMDQGEYGVGSGRSQQQNLGGVGAGSGQSGGDTYDD 197

R G GL + D YG G T G + G+D GS N G+G+ + D Y

Sbjct 519 RNGQSGLDSSDTYG-SGNTSGRTGGLDSTSDTYGS-----SNTDTYGSGNTNTSSDNYGS 358

Query 198 GN----SGGKK--DSTMGKLMEKAGGMLKNEKLEQKGAQKRAEQGGYDD----TTGGSGG 247

GN SG K DSTMGKLMEKAGGMLKNE L++KGA KRA+ GG D+ TG +G

Sbjct 357 GNQNTSSGNNKKNDSTMGKLMEKAGGMLKNEGLQEKGAAKRAQAGGNDEYSSGNTGNTGS 178

Query 248 YGGDNTSSGY 257

YG N + Y

Sbjct 177 YGSGNNNDNY 148

***Agave deserti* (accession GAHT01100705)** (E-value: 5e-07)

Query 1 MSY--NDDSSRGGQYGSSNTGGGMGQDDSYGSSGRTG-GAQGTDDSSYGTGAQGGMRQGG 57

MSY NDDS YGSS +DSYGS G TG G+ +D SYG+ R+

Sbjct 53 MSYRGNDDS----NYGSS-----RNNEDSYGS-GTTGFGSSRNNDDSYGSTT----RRDD 190

Query 58 DDSYGSSRQGNT----GGGLGQDDTYG--SSRQTGGAQDDSYGSS---GRAGGATGGGLG 108

+DS+GSSR+ N + ++G +S GA SY +S GR G

Sbjct 191 EDSFGSSRRDNEDSYGSSNPVKHSSFGDSTSSNLAGADSLSYNASENKGRTYDNDPTSYG 370

Query 109 ADDTYGSSNQSGGAQRGGYGGDDTTSGGSKRTGGGLGA-----DDNYGNEGATGGASSGM 163

++D+YGSS ++ + +G +T G++ T DD+YG+ G GGA +

Sbjct 371 SNDSYGSSRRNDDDE-DSFGS--STRRGNETTTSSTYGTTGRNDDSYGSSGRVGGAVN-- 535

Query 164 DQGEYGVGSGRSQQQN-LGGVGAGSGQSGGDTYDD--GNSGGKK-DSTMGKLMEKAGGML 219

D YG SGR + + G + S DD G+S K DS +GK++EKAG ML

Sbjct 536 DS--YG-SSGRRDENDSYGSSRYDNSSSSRRDNDDSYGSSSDKGGDSKLGKVLEKAGSML 706

Query 220 KNEKLEQKGAQKRAEQGGY 238

+EKL+ KG +KR +G Y

Sbjct 707 HSEKLQNKGYEKREAKGAY 763

***Lolium perenne* (accession GAYX01124046)** (E-value: 4e-06)

Query 16 SNTGGGMG--QDDSYGSSGRT-----GGAQGTDDSSYGTGAQGGMRQGGDDSYGSSRQGN 68

+NT G G +D+ GSSGR G G+D SYG+G + D+YGSS

Sbjct 12 TNTSSGFGSSNNDNLGSSGRNTDTYGSGNTGSD--SYGSGN----KTSSSDNYGSSNTDT 173

Query 69 TG-GGLGQDDTYGSSRQTGGAQDDSYGSSGRAGGATGGGLGADDTYGSSNQSGGAQRGGY 127

G G D YGSS G+ DSYGS G + G D+YGS N++ + Y

Sbjct 174 YGSGNKSSSDNYGSSNNNTGS--DSYGS-GNKSSSDNYGSSNTDSYGSGNKTSSSDN--Y 338

Query 128 GGDDTTSGGSKRTGGGLGADDNYGNEGATGGASSGMDQGEYGVGSGRSQQQNLGGVGAGS 187

G +T + GS G + D YG +T ++G D YG S N G+G+

Sbjct 339 GSSNTDTYGS----GNKSSSDAYG--SST--TNTGSDT--YG-----SSNTNTDSYGSGN 473

Query 188 GQSGGDTY---DDGNSGGKKDSTMGKLMEKAGGMLKNEKLE 225

S D Y + N KDST GK+MEK GGMLKNE L+

Sbjct 474 KTSSSDNYGSSNTDNDSKSKDSTTGKIMEKVGGMLKNENLQ 596

***Triticum polonicum* (accession GEDP01247456)** (E-value: 1e-08)

Query 9 RGGQYGSSNTGGGMGQDDSYGSSGR-TGGAQGTDDSSYGTGAQGGMRQGGDDSYGSS-RQ 66

R GSS T G G+D+ +GSS R G+ D+ +G+ + G D++GSS R

Sbjct 704 RTENIGSS-TLGSSGRDN-FGSSDRDNFGSSDRDN--FGSSDRR-TENIGSDNFGSSGRT 540

Query 67 GNTGGGLGQDDTYGSSRQTGGAQDDSYGSSGRA--GGATGGG--LGADDTYGSSNQSGGA 122

N G G+ D +GSS +T D++GS+GR+ G++G L +D TYGSS ++G +

Sbjct 539 DNIGS-TGRSDNFGSSDRT-----DNFGSTGRSDNFGSSGRNDDLTSD-TYGSSGRTGAS 381

Query 123 QRGGYGGDDTTSGGSKRTGGGLGADDNYGNEGATGGA-SSGMDQGEYGVGSGRSQQQNLG 181

G G G S RT DN+G+ G T SSG + YG + R NL

Sbjct 380 DTLGSTGRTDNFGSSDRT-------DNFGSSGRTDNFGSSGRNTDTYGSDNRR--NDNLS 228

Query 182 GVGAGSGQSGGDTYDDGNSGGKKDSTMGKLMEKAGGMLKNEKLEQKGAQKRAEQGGYDD 240

G + GK DSTMGKLMEKAG LK +L++KG +KR EQ GY+D

Sbjct 227 GSSYDNDNKNN--------NGKGDSTMGKLMEKAGAALKKPELQEKGHEKR-EQKGYND 78

***Sorghum halepense* (accession GGDZ01001819)** (E-value: 4e-09)

Query 13 YGSSNTGGGMGQDDSYGSSGRTGGAQGTDDSSYGTGAQ---GGMRQGGDDSYGSSRQGNT 69

+G S+ G G ++ SYGS+ RT S G G R+ DDS+GSSR+ +

Sbjct 172 FGDSSYGSGRTENQSYGSN-RTENESSFGSSGMGVSGSDSYGSNRRDNDDSFGSSRRTD- 345

Query 70 GGGLGQDDTYGSSRQTGGAQDDSYGSSGRAGGATGGGLGADDTYGSSNQSGGAQRGGYGG 129

+G D+YGSSR TG S G++ + + G D+T+ SS G YG

Sbjct 346 --NIG--DSYGSSRNTG-----STGNTYGSSDSYGSSRRDDNTFSSS---GRTDNDSYG- 486

Query 130 DDTTSGGSKRTGGGLGADDNYGNEGATGGASSGMDQGEYGVGSGRSQQQNLGGVGAGSGQ 189

S G + G+G+D NY + T G+ +G D YG SGR Q GVG+ +

Sbjct 487 ----SSGLQ----GVGSD-NYSSSN-TYGSGTGTDS--YG-SSGR---Q---GVGSDNYN 609

Query 190 SGGDTYDDGNSGGKK-DSTMGKLMEKAGGMLKNEKLEQKGAQKRAEQGGYDD 240

+G D+Y G K DSTMGKLMEK GG LKN+KLE++G +KR EQ G +D

Sbjct 610 TGSDSYGSGRQDKSKGDSTMGKLMEKVGGALKNDKLEERGHEKR-EQKGLND 762

***Cordyline australis* (accession GFHQ01026283)** (E-value: 2e-09)

Query 9 RGG-QYGSSNTGGGMGQDDSYGSSGRTGGAQ--GTDDSS--YGT-GAQGGMRQ--GGDDS 60

RG YGS NTG DSYGSS RTG + G +DSS YG+ G GG GG+DS

Sbjct 202 RGNDSYGSGNTG-----SDSYGSSNRTGDSSFGGKNDSSDTYGSSGRTGGDSSFGGGNDS 366

Query 61 YGSSRQGNTGGGLGQDDTYGSSRQTGGAQDDSYGSSGRAGGATGGGLGADDTYGSSNQSG 120

YGSS + N D+YGS GG +DSYGSSGR + G G D+YGSSN++G

Sbjct 367 YGSSGKNN--------DSYGS----GG--NDSYGSSGRGNDSYGSGNTGSDSYGSSNRTG 504

Query 121 GAQRGGYGGDDTTSG---GSKRTGGGLGADDNYGNEGATG---GASSGMDQGEYGV-GSG 173

+ G G + ++SG S TG G G+ D G G G G+S G YG G+

Sbjct 505 DSDTYGSGQNKSSSGQDYSSGNTGSGYGSSDRTGGSGLGGDSYGSSDNTSSGNYGSSGNN 684

Query 174 RSQQQNLGGVGAGSGQSGGDTYDDGNSGGKKDSTMGKLMEKAGGMLKNEKLEQKGAQKRA 233

+ + G SG D ++ SG DST GKL+EK GG+ K+E L++KGA KR

Sbjct 685 NTSSDSYSSNDNSYGSSGND--NNNRSG---DSTAGKLLEKVGGVFKSEGLQEKGAAKRD 849

Query 234 EQG 236

G

Sbjct 850 AAG 858

***Panax notoginseng* (accession GFRX01134804)** (E-value: 1e-31)

Query 11 GQYGSSNTGGGMGQDDSYGSSGRTGGAQGTDDSS------YGTGAQGGMRQGGDDSYGSS 64

G+ G+ +GG DD+YGSSGRTGG G DDS+ +G+ + G G D +GSS

Sbjct 233 GRTGAQASGGYGASDDTYGSSGRTGGGLG-DDSTGRRGDDFGSSGRTGGGLSGGDDFGSS 409

Query 65 RQGNTGGGLGQDDTYGSSRQTGGA---QDDSYGS-SGRAGGATGGGLGA-DDTYGSSNQS 119

G TGGGLG DD++G S +TGG +D+ YG SGR G GG GA DDTYGSS ++

Sbjct 410 --GRTGGGLGADDSFGDSGRTGGTSTGRDNEYGMGSGRTGAQASGGYGASDDTYGSSGRT 583

Query 120 GGAQRG-----GYGGDDTTSGGSKRTGGGLGADDNYGNEGATGGASSGMDQGEYGVGSGR 174

GG G GDD G S RTGGGLGADD YG+ TGG S+G D +GVGSGR

Sbjct 584 GGGLGDDTTGSGRRGDDDF-GSSGRTGGGLGADDTYGSSTNTGGTSTGRDD-NFGVGSGR 757

Query 175 SQQQNLGGVGAGSGQSGGDTYDDGNSGGKKDSTMGKLMEKAGGMLKNEKLEQKGAQKRAE 234

+ QQ GG GAG +TYDD NS KKDST GKLMEKAGG+ K+ KLE++GA+KR

Sbjct 758 TDQQQTGGYGAGD-----NTYDDDNSSNKKDSTTGKLMEKAGGLFKSSKLEERGAEKR-R 919

Query 235 QGGYDD 240

Q G DD

Sbjct 920 QAGQDD 937

***Nepenthes khasiana* (accession GEXD01067829)** (E-value: 9e-19)

Query 2 SYNDDSSRGGQYGSSNTGGGMGQDDSYGSSGRTGGAQGTDDSS---YGTGAQGGMRQ--- 55

SY DD+S GG G S GG GQDDSYGS GA+G D S+ YG+ + R

Sbjct 61 SYGDDNSYGGS-GRSGKTGGYGQDDSYGS-----GARGDDTSASTGYGSNTRAHERSSGL 222

Query 56 GGDDSYGSS-RQGNTGGGLGQDDTYGSSRQ-TGGAQDDSYGSSGRAGGATGGGLGADDTY 113

GG D+Y S+ +G G D+YG + +G QDDSYGSS R G TGG G + Y

Sbjct 223 GGSDNYSSTAMEGQPRSGRNTGDSYGDNNLGSGYGQDDSYGSS-RTGRNTGG-YGDSEDY 396

Query 114 GSSNQSGGAQ--RG---GYGGDDTTSG-GSK-RTGG----GLGADDNYGN-----EGATG 157

S+ +G RG YG D+ +SG GSK R G GLG D+Y + + TG

Sbjct 397 SSTAMTGQPTSGRGQADSYGQDNLSSGYGSKTRRGNEQSSGLGGTDDYSSAALEEQPRTG 576

Query 158 GASSGMDQGE---YGVGSGRSQQQNLGGVGAGSGQS-GGDTY-DDGNSGGKKDSTMGKLM 212

G + D +G+GSGR+ Q GSG+ D+Y DD K DST GKLM

Sbjct 577 GDYASADTARDDAFGMGSGRTADQE------GSGRRYDDDSYGDDNKQSSKNDSTTGKLM 738

Query 213 EKAGGMLKNEKLEQKGAQKRAEQGGY-DDT 241

EKAG +LKN+ LEQKGAQKR E GGY DD+

Sbjct 739 EKAGSLLKNKNLEQKGAQKRGEAGGYGDDS 828

***Colobanthus quitensis* (accession GCIB01005368)** (E-value: 7e-14)

Query 6 DSSRGGQYGSSNTGGGMGQDDSYGSSGR---TGGAQGT-----DDS-SYGTGAQG--GMR 54

D++ YGSS + DSYGSSGR G+ T DD+ SYG+ + G

Sbjct 1055 DNTSSDNYGSS----RKNESDSYGSSGRDQDNLGSSNTRSSNRDDNDSYGSSKKDTYGSS 888

Query 55 QGGDDSYGSSRQ--GNTGGGLGQDDTYGSSRQ---TGGAQ--DDSYGSSGRAGGATGGGL 107

+D+YGSS++ NT G +D+YGSS++ T G+ DDSYGS + + G

Sbjct 887 NNDEDNYGSSKKDSSNTYGSSNNEDSYGSSKKDTNTFGSSNVDDSYGS--KKDSSNTYGS 714

Query 108 GADDTYGSSNQSGGAQRGGYGGDDTTSGGSKR----TGGGLGADDNYGNEGATGGAS-SG 162

+D+YG N+ + G DD G SK+ T G G DD+YG++ + S

Sbjct 713 NNNDSYG--NKKDSSNTFGSSNDDNEYGASKKDSSNTYGISGNDDSYGSKKDSSNTYGSS 540

Query 163 MDQGEYGVGSGRSQQQNLGGVGAGS----GQSGGDT---YDDGNSGGKKDSTMGKLMEKA 215

D YG S R + +G+ G S +T Y D NS GK DST GKL+EKA

Sbjct 539 NDNDTYG-SSKRDNDNSNTYYSSGNTDSYGSSDRNTSSGYGDNNSSGKGDSTAGKLLEKA 363

Query 216 GGMLKNEKLEQKGAQKRAEQGGYDD 240

GGM KNEKL QKG +KR+ GYD+

Sbjct 362 GGMFKNEKLTQKGQEKRS-NAGYDN 291
