# Supplementary material for: TAS3 miR390-dependent loci in non-vascular land plants: towards a comprehensive reconstruction of the gene evolutionary history
Source: PeerJ. 2018 Apr 16;6:e4636. doi: 10.7717/peerj.4636 (PMC5907777; doi:10.7717/peerj.4636)
Supplement: Figure S3 [file peerj-06-4636-s003.doc]

**Figure S3.**

**Nucleotide sequences of TAS3 loci in plants from Anthocerotophyta and Marchantiophyta.**

Nusleotide sequences complementary to miR390 are in yellow.

***Folioceros fuciformis* (accession SRS2162762)**

GGCGTTATCCTTCCTGAGCTGAGAAAGAAGGCAAGGGTGGGGGTGGCGTGGCGGGCGGCGCCTTGTTAACGGGGTGTTAAGCACCAACGGACGCCCTGGCAGCCTCAGACGCCACCCACGGCTCCGTAGGGTGTGATGAGTGCTTTACCTAgcgctcagcccctggcgagcccacctacccttgtgacacgggcctggcagatccctgcacggcccctgtcggttacgtatcactcctgagcta

***Marchantia polymorpha*** 1-Mpo **(accession** [**KC812742**](https://www.ncbi.nlm.nih.gov/nucleotide/516429953?report=genbank&log$=nuclalign&blast_rank=1&RID=7UUET1BJ01R)**)**

GACGGTATCCTTCTTGAGCTAAAAAGATGTAGCTTCCTGCTACATCTCACACGACACATCTCATTTGAATGTTCAAATCTTTAGTGACTGAATCGAATACTAAAGTTAATTTGACTTCAATAGAGACTAGTTTGCGGGAGAAACTGTGCCAGTTAGCAGGAGGGTGTGATGAGTGCTTTACCTGGTCCAGGATCCCCACCCCCTCCTCCACTGCCTATTTCTAGGCTCGCGTTACCTGCCTATCCCTCTTGAGCTA

***Marchantia emarginata* (accession SRX1952816)**

GACGGTATCCTTCTTGAGCTAGGAAGAAGGAGATGTAGCTTCCTGCTACATCTCACACGACATGTCTCGTTTGCATGTTCAAATCTCTCGGATGTAAGTCACATACAGAAGTTAATTTGACGGCAAGCGAGACACATGTGCGGGACGGACACCCCTGGTTAGCATGAGGGTGTGATCAGTGCTTTACCCGGCTCGGGTTCCCGTCCCCTTCCCCCACTGCCTATGTCTAGGCTCGCCTGACCTGCCTATCCCTCTTGAGCTA

***Conocephalum japonicum* (accession SRX1952810)**

GACGGTATCCTTCTTGAGCTAGGAGGAAAGAGATGTAGCGAGCGGGACCTTACCTGCTACATCTCACACGACGTGTCTCGTTTGAATGTGCATGTCACAATTTAATTGGATGATCAACGAGATAAATGTGTTGGATGGACTCTCCTGGCTAGCATGAGGGTGTGATGAGTGCTTTACCATACAGGGGTTCCCGTCCTTTCTTCCCGTCGCATATGTCTAGGTTCGCCTGACCTGCCTATCCCTCTTGAGCTA

***Ricciocarpos natans* (accession ERX337127)**

GACGGTATCCTTCCTGAGCTAAGGGGACAGGAAAATCTGCTACATCTCACACGATACGTTTCTTTGGTATCTTCAAATCAGAATATGATTCGAAGATACACGAGATAAATTTGTGATACGGACACCTCTGCCTAAAATGAGGGTGTGATGAGTGCTTTACTAGGCAGGGGTTCACGTCCATTTCTCCCACTGCATATGTCTAGGTTCGCCTGACCTGCCTATCCCTCTTGAGCTA

[***Dumortiera hirsuta***](https://www.ncbi.nlm.nih.gov/sra) **(accession SRX1126014)**

GACGGTATCTTTCTTGAGCTAGGAGGAACGAGATGTAGCTGTTCCTGCTACATCTCACACGACATGTCTCATTTGTATGTTCAAATCAAAAATTAATTTGACAACACACGAGACAGACGTGAGGGACAGAGACCTTTTGCTAGCATGAGGGTGTGATGAGTGCTTTACCAGGCAAGGGTTCACGTCCTTTTCTCCCATTGCCTATGTCTAGGCTCGCCTGACCTGCCTATCCCTCTTGAGCTA

***Plagiochasma appendiculatum* (accession SRX1741567)**

GACGGTATCCTTCTTGAGCTAGGAGGAAAGAGATGTAGCGTTTAGTGCTAGCTACATCTCACACGACATGTCTCGTTTGTGTGTTCAAATCAGAAGTCAATTTGATGACAAAAGAGACAGACGTGTGAGACGGACGCCTTTGGCCAGCATGAGGGTGTGATGAGTGCTTTACCAGGCCGAggttcccg

tccttttctcccattgcctatgtctaggctcgcctgacCTGCCTATCCCTCTTGAGCTA

***Conocephalum conicum* (accession ILBQ_2006554)**

GACGGTATCCTTCTTGAGCTAGGAGGAAAGAGATGTATCAAGCGGTGCCTTACCTGCTACATCTCACACGACATGTCTCGTTTGAATGTGCAAGTCACAATTTAATTCGATGATCAACGAGATAAATGTGTTGGATGGACTCTCCTGGCTAGCATTAGGGTGTGATGAGTGCTTTACCATACAGGGGTTCCCGTCCTTTCTTCCCGTCGCATATGTCTAGGTTCGCCTGACCTGCCTATCCCTCTTGAGCTA

***Lunularia cruciate* (accession TXVB_2071521)**

GACGTTATCCTTCTTGAGCTAGGAAGAAGGAGATGTAGCTTCCTGCTACATCTCACACGACATGTTTCACTAGAAAATCTAGTAGTGAAACAATTGTGTGGGACAGGCCTGCCTGGTAAGCACTAGAGTGTGATAGGTGCTTTACCAGACGGGGGCTCCCGTCCCCTTCCCCCACTGCCTATGTCTAGGCTCGCCTGACCTGCCTATCCCTCTTGAGCTA

***Metzgeria crassipilis* (accession** [**ERX337128**](https://www.ncbi.nlm.nih.gov/sra/ERX337128%5Baccn%5D)**)**

GACGTTATCCTTTCTGAGCTAGACAGACGTGACAGGATGTATCTGGCTACTGCTGCATCTCACACTATGGGAGGGCTGGCGAGCTGGAGCTGTTCTCAGCCTTTATGGTGAGATACCCTTGGTCAGCTTTAGGGTGTGATATGTGCTTGACGTGGCAGTCTCACCCGCGCCCCTGGGCAAATGCCTGCATCTTCCTATCTGTTAATTTCCTATCCCTTCTGAGCTATCTTGGcggcatccctcctgagctt

***Pellia endiviifolia* (accession SRX726500)**

GACGTTATCCGTCTTGAGCTAGATAGATGTGGTTCTGCTGCATCTCACACTACATGCGGCCCTCTCTTGCTGGCAATGACAACTAGGTGAGCATGGAGGTGTGATATGTGCTTGACGTGACTTTCCCTCCTGCCCCTGATTTGTTTTGCCTGTTGCTAGGCTAGTCTTGTATCTCCTATCCCTCCTGAGCTA
