# Supplementary material for: TAS3 miR390-dependent loci in non-vascular land plants: towards a comprehensive reconstruction of the gene evolutionary history
Source: PeerJ. 2018 Apr 16;6:e4636. doi: 10.7717/peerj.4636 (PMC5907777; doi:10.7717/peerj.4636)
Supplement: Figure S4 [file peerj-06-4636-s004.doc]

**Figure S4.**

**RNA transcripts of the selected Bryophyta TAS6/TAS3 loci found in 1KP database.**

***Takakia lepidozioides* (accession** [**SKQD-2076588**](http://www.bioinfodata.org/Blast4OneKP/search?Sequence=SKQD-2076588)**)**

**TTTATGATGAAACAAATCCATCTTGATTTTTACTATACCAGTGGAACATGTATGTTTGTTTAGTCTGGCAGCTAACACTAGGCAGCTGCCAATGAGCAATGATACATGCTCGAAGAATCAGCTGAAATTCAAATAGTGTAAGATTTGATACTACAACTTCACCTATAGTTATCCCACCAAGTGAACATTGAAAGACTCGAAATTAATAATGCCACTCTAATAATAATTAGTAATAGGGGCAAGATTTAGCTCAGGAGTGATAAACAAATTTACAAGGATGGCGCAAAAAGTTGCAGTCCCCTTGTACCAAGGGTAGGACGGCTGGGCCAATGTGAGGTGCTGGTAAAGCACTCATCACACCCTAGGCCACCATATCCGGCACAAGGCCGGGAAGTGTCCTAGTGCCCCCCCTCAACCCACCCTCTACTGGCTTAGCTCAGGAAGGTTAGCGCCTGTGGTGGATTTAGATCAAAGAGGCAAATTCAGCCATCATGATGGGGTTCGATGAAGATGTTGAAAGACTTGTGTATTTGTGTGTTGTTGGAGGGACTGGAGGAAGTCTGATGAAAGATTTTCCTGCAGAGATGCTCAAATCTGCACACACCATTGCCACGGGGTAACAAAGGCAGCTTGAAGAAGTATCCTCCACTAGAGCATGTCTTGATGGAAGATTGTTGATTACAGCAACCAGAGTCTAAGCAAACCTACTTCTGATATCCCTGGATTGACGTGATCCTCGATAACACTGGAAATAGGCCAGCCACCAACCTCCAGTTCACTTAGTATTCAAGCTCCGACGAATAGAAAGTCACAGACACGGCCTCCGCCCACTAGTTGTCCTCCACCGTACAGCTCTAGGCTCTTTATCAGGTGGACCTCCGGAAACGCCCAAGGATTTATCTTGTCGCCCCCCGATGACTGTCGATCGTTCGACAAATGCTCTCCAAACGCTGGTTAATCTGAAGCGAGAGAGCACTATGCGAGCTGCCAATTGCACGAAGGAGACAGTTCTAAACGGTTTCACTCACAACCAGACATTGCAGGTATCGCGCAGAATGGAAGTCCGACCGGCCCAACTCATGGAAAATGCAAAGGTCTAAACAAACAAACAAGCGCGGGCTGCATAGAGGAGAACTGTAGTGCTATACACACTAGAGCTGCATGACCTCGCTACAGTAGAATAGAGACCTACCGTTTAACCAACTGAAAGTCACTAGAGAGCACGTAGCTACAGGAACAGAGCGGTGGAGCAAGTGTGGCGAGAGAAGAGAGGGAGAGAGAGCGCAAGGCCTGGAGAGCTGCGGTGTATTGAGTGCACGCCTATACCAGC**

***Andreaea rupestris* (accession** [**WOGB-2010369**](http://www.bioinfodata.org/Blast4OneKP/search?Sequence=WOGB-2010369)**)**

**GGAGCTGATTGTGCTAACCACTTGGAATGACCTTGTTCTTGGAGCTGAATTTTGCGCTAACCAAATGTAAACATTCAACAACTTCTCTTTGAATGACCAAACAATCTCTACTAGCTCAGGAGTGATAATCGACCTGACAAGGGGCGCATAGTTGCCTATACCCCTCTTGTCCCAAGGGTAGGTGGGCTGGGTAACGTAAGGGTGCTGGCTAAAGCACTCATCACACCCTAGGGAGCCATAGCTGACGTCTTGCGACAGGGTCTCGCACTAAAGAACTCCCACCCCACCGTCAAGGAATCCACTACGCCTACTAGTGCCCCGCGACCAACCTCCCCCTTGCTTAGCTCAGCAGGGATACCGCCCACCAATCCTCTACGCATGTGTCAAAAATTCACCGGCGACTGTCCAAGGTTGAGAATGTCTGGTTGCAGCAAAGAGATAGAGGTGAGCTGGGATGTAGTAAAGAGAAGGAGGGAGCCTGATGAAAGACTTTCCTGCAGAAATGCTGAGGTCAGAGCACACCATTGCCACAAGTAGAATCTACTGCCCTGTGTCAGCAGAGCATGTTTCACTAACCAGGTTGATTGCTGAATACTCTCAGGACAAGAAACAAGGACAATTTCTCTGACTAACGTCCAACTTTGATCATCTGTAGATGCACTTGCTGTTGGTTGATAGATCCGTCACTGTGAGTGGCCTTGTAGGAGTTCTGAGTACTTCAGTGTTCCTTCAATCGAAGTTGGAAAGTCTGTCCAACCTTGCTGGCCTTCTAGATCAAGAGATATTCGACTTCGAAGCTCTCCAGTAGGTCAAGAGATGAGTGCACATCAAATGGATTTCGAACTTCACAGGTTGTCGATGCAAACGGAGGCGATTCAATGTATTTGGGAATTGACGAGACTGTTTTCACTGATATTTAAGTGGATTCAATCAGTTGGGTCTAGTCACAGAAGAGTCTTTTGACAGAAGAGAGCGAGCACGGCCACTGAAGAGATGGGGATCGAGCACACACAGCAACCCACTTAAAGAACTTGACAGAAGATTTTTAAAGGGTTCCCGATCCAATATCTGCGTCAAGACACAGGCGTACAGGAAGCTGAGCGCCTAGATAAATGACTTTTACTTGTCGGTACTCTGGCTAAAGTAGTACTTTTAATCGCAGAAATGAAAACAATAATTAGATAAAATCAGACTTCAGGACAACAGCTTGGATCAATCCTACTTCAATGCAGGGAGACTAGTTGGATGAAAGACCTCGAATGGTTTGCAATTGCGATGAAGGAACTGCAATTCTGTATACACAGGATTTGGTACTCTGTCCCCTTTGACCGTGATGAGAGTAAGCAGAAGAAACTGGATACTCCGAAGGCGTACTTATGGATCCAAAGGGAGTGTCAAGAACAAAGAGGACACAAACTCGCAAGCACAAGGAAGGTTGTTAACTTGAATACCAAGATACTTGACCTACGACGGCAACGTAGGAAGTCACCGGACGAATAAAAGAAAAAATCCCGTGCAAAACTCGAAGTCAAACCGAGCCTCAGTCACCAGCTAAAGTGCAGGACGGTACTCGTGCGAGACTGCTGAAACACAACATAGAGAATATCGATTCAAGCACACCGTCACCTCTCAACAACCAAGCTGCACAACACCGAACAAGCGCGCATGTTTCAAAACAGAGGAAACGAGAAAACAGAACCTGGAACAGTAAATAATAAGAGTGAGCTGGGAAGTATGTAAAAGAGTCGCTACATAGCAATAATAGATGAGTAAATTCCTAGACAAGGGAGTGGACACTGTGAGCTCCCAAAAACCGACACAACCCAACGGTAGTCGCCCAAAAGTTCAGCAGGAGTACGCAGAAGAGGGAAGTGAGACACCACACTACTAGAGACGAGAGCTGCTCCAGCAGATGAGGATGAATGCAAGTGGAAGAGGGAAGCTGGGAGCAGTTTAATAGGGGATCGACCGCCCTGTCGACTGCTTTCTATGAATGTGCC**

***Atrichum angustatum* (accession** [**ZTHV-2082998**](http://www.bioinfodata.org/Blast4OneKP/search?Sequence=ZTHV-2082998)**)**

**AATGGAATTACTTGTTCTGGAATACGTGAAGGTGTTCTTGTAACAGGACTTGCCGTGTTGTTCTTGGTCCCATCGCCGAGCAAATTTGAGAGTTCTTGTGCGAACAGCTAGTAGTCTCCGAGATGAGAGGTATCTTGGCGACGCAAGGGGAGCCACAAAGATGGTGTTTTGGTGTTGTAGTGGCAGATTAGTGCCCTTTGGGTTGCCTTGTTGTTGTTGTTGTTTTTCTTGGGGTGTAACTGGGCCTATCCTAGCGACAGTTTCGGTGTTTCCAGGTGCTTTAGTGTTCAACAGCTTTCAAGGTTTCGTTTTCTGAAGGTTAGCCAGCAGTACTGTAGTCGTCGGTGTCCAAACCTCTCTAAATTCAGTTCTTCCCAACACAGCTACGGTTTGCATCTTTGTAGTAATTTGTCATTACAGTTTCGCCTTTGGCAGACAATCTGGCTTTTGTCAAGAGCCCTCGTGGCTATGGTGTAGCTCACATTGCAGCTGTGCTCCCTCACATCTCTTCAGTACTGTTTAATTCTCGCCAGTACATCTCTGCGCTGCAATTCATCTGTGGAGGGTTAGGTGCTAAAGGTTTCTGTCCCCCATCAAAACACCAGGGTTGTTATAGTTGCCAATCTTCACATATTGTAGAGGGTGGAGTAATCTGATACCAGTCCTGTGTTCACTCTTTTCTGTCAAGCCACGAATCCCAACGGTGGGATTACCCGTGGGCATCACTATCCATTTGCTTACTCATCTAAGCAACCAAAGGCAGGGCTATCATCCCCGAATCTGTGGAGGAAGGCGAAATTCTCTTGAAGACACTGTCTGTTGAGGTTCATGCTGAGGGTGGCTTGAAGATAGCCACAAATTGCGAATTAATTAATTAGCAGAGAGAAGTCATGGGACTCTCATCTGGGAAAACCAGGATGATCACTGGGCCAGCCCCTGGAATGATCTTATCTTCACCAGCGGACCTCCTCCTTGTAGATCGAATAAGAAGTTCGAGAAGAATATTATTGGTGATGTCGATAGTTGTGCACTTAGACTCTCTGGCTCATGCAATGTATAATTAGGTTTCTGTTCGGCGGTATCCGTCTTGAGCTAAGTTAAGCAAGGGTTAGGGCTGGGCTTGTGGGCGGGGTGCTAGGCACTCCGGTGCGAGCTCCGTCCTAAGACGTCAGCTGGGGCTCCGTAGGGTGTGATGAGTGCTTTACCAGCTACTCATCACCTGCCCAGCCCACCTACCCTTGGGACACGGGCTGCGTGGATTCATCCTGAGCCCCTGTCCGTTGTGTATCACTCCTGAGCTAAGTAAGTAGTGCACCCCGTCCGCCTCCGACCTCGTCCTGGAAGCAAATTGATGAGAGATTCGATGATTGTATGAGAGGATCAAATTCCCTCAGAAATTAGATCTTGGGGCTGCCACTACAGGCATGCGCCATGCCAACTTGTCGGTACAAGCAAATCTTATATTATAATCAAAAGCAAGCAGCACGGTCTACC**

***Tetraphis pellucida* (accession** [**HVBQ-2019753**](http://www.bioinfodata.org/Blast4OneKP/search?Sequence=HVBQ-2112923) **)**

**CTCAGGAGTGATAATCAACTTGACAAGGGGCTCGTAGAAAGCTACGGCCCACTTGTCCCAAGGGTAGGTGGGCTGGGCAGTGTGCGAGGCTGGCGAAAGCACTCATCACACCCTAGGGAGCCATAGCTGACGTCTTGCGGCAGGAGCCCCCCATGAGGAGGTCCCACTCCACTTTCATGGCGGCAGCTATGCCTACTAGTGGCCCGCAACCAACCTCCCCCTAACTTAGCTCAGGAGGGTTACCGCCCAAATTTCCATTACATCTCTCTAGCAGATTCACTGGCACATACAAGCCTTGTGTATGCCCTGTCAACAGTTGTACGNNNNNNAGAACACCTTGGAGAGCATGGCAGCGAGTTGGAGAGATCGCCTGCCATCGCCAAGAGGATGGAGTGCACCATCACCATTTCCCAGGATCGGACTTCTATGCTTAGGATAACAGAATATCAGGAGGCTGGCGAAGTGGTGTCAAGAAGCATAGAGAGACACTCCTTGAAAATTCGTAAGCCTATGATGTGCCCAAGTAGTTGCAACTCAATTCAATTCGTCCTACCTGCACCCTTCTGAAGTTTCCTGGGAGGCATGGATCGAGGAAGGAAGGTCCAAACTGCGATTTCGCCCTTCGTCAGACGACATAAAATCTTCCCTGCCTTCGTTCGAGGAAGACGCGTCAGATACAGTCAAGCAGCACCTGCCAAGGGTGGTACTCAGACGTCGGGAGTCTGCTGGTAGGATCGGGGTGCACACTGCAAAGTGGCAAGCTCTTGACAGATGAGAGTGGGCACAGAGGACTGAAGAGATGGCGATGGAGCACAGCAGCCGAAGTAATGAATCTTCCACTTGGTAGGTCCCAAAATCACTTTCTTGTAACTGCAGGGTCGAAGAGAACACAGCTCAATGAAATGCAATTTCTGTTCAGTGATCGGAACCACACTGCTTAGAAACGATCGGGTGTAACAATCTTCTCGACAAAGGACAACAGAAAAATGGTGCAACAACTTCTGCACCAATTTTATTGATGGCTAAGTAGTGGAAACGA**

***Diphyscium foliosum* (accession** [**AWOI-2069791**](http://www.bioinfodata.org/Blast4OneKP/search?Sequence=AWOI-2069791)**)**

**GTCTATTACTTCCACTTAGTTCTTTGTTGTTTGCCGTTAGCACCATTGTGCTCCCCACGTAGGAGGGCCAATCTCAAGTTCCCTTTACCAAAGTAGGTATATACAGGGCCACTGAAATTTGGACAATAATGACCACAATCAGGCTCAGTGGGTTCTTAAGCACATCACCTACTTGGAAATGATCTCTGCAATCAGAAGCGCCCAGATTTGCCGCCCTTAGCGATGCTCTGTCATTGATACACTAACTTTCACATTCGTAGCTCATCTGTAAGCGTATCAAAGCGGCTGTGCTCACTCACCAACTCTTCAGATGTGTTCTCTCTTCTCACTGTCATGACCCCACTTCCTATCTACATTCAACACAAAGGCTGAGGCCAGGATCTTTTCACTCTGGACCCTCGTTGGGAATAAGAATCCATGTTGCTTCGCGATTGACAATCAGGGATACTGCACTATCACTACTGTCACTCTCCTCTCTGAAGACCAATCTGCCGAAACTGAACTGTCGACAGTCCTTTCACCCGAGCTCATGCATCGGTGGAGGGAGGTCCAGCTCGCAAGGTGCTGAGGATATATCTGCTCCGATTTGTGTGTTGACGTCAGTGGTTGAGTGATTTCAGGACTGGAGCTGTCCATCATATCGCATCTCTGCACTGGATTTGGTGTCTGGAACGCTGGCCGCTCGACATAGGTGATAACTCTAATTTGATCTCTTAAGACGTAGGCACTTTGTTGCAATGGTGTGCTCAGATCTCTGCATCACTGCAGGGAAATCTTTCATTTGGGTAGCTACACCCCAGTCATGCATTTTAGCTCAGTTCCTCTTTGGAGATAAGAGTGAACTGTGTCCATCGATGGTGGATGTAGTCACTTTTCTTGTATAGAGTACCTTCAGGCGGTATCCTTCCTGAGCTGAGAAAGAGGCCAAGGCCCTTAGGGCAGAAATAGGTGAAGCTGACGTGTTGTAGACTGTGTGTTAGACACATGAGTGTAACACATCGGGCTAAGACGTCAGCTATGTCTTCATAGGGTGTGATGAGTGCTTTACCCGACGCTCATCTACTGCCCAGCCCACCTACCCTTGGGACAAGGGCTGTGCAAATTTTTGTGCGGTCCTTATCGGTTGTATATCACTCCTGAGCTATTGCATTCATTTCTCTTACCGG**

***Fontinalis antipyretica*** **(accession DHWX_2007057)**

**CTAGCTTGGGAGCATGCTCCTCTTCCTCCTCAAGGCATGCAAGGGAGGATACGTCTCGCTTCTCTGGACAGCTTCCGTCTTGGGCCGGCTGGCTCGATCACGATGACGATGGGGCATCGCATCGCTCTCCCGGCTGTGTTCTCACAGGTTTCGTGGATAGCGAGCCTCTTGGGATCCTAGCTCGTTCGTTGAGTGAGTGAGTGAGTGAGTGAGTTGAGTTAGTTAGTTGGTTGGTGGCCGGGCGTCGAGCGAGTGATTTTGCTTTCTGTCGAAGTTTTGGGGTTCCAGGGGTAGCAGTTGGTGCATGACCGTGTTTTTGTTTTGGGGATTTTGTTCCAGGGTTAGTGAGAGTGAGAGTGATCAGTTGGTAGGGTTCAGGCAACACGTTAGAGTCCGACTGACTTTACTGCATTTGATCAGGGTTTGCCGCATGTGTTTGGGCTTCCAAGGTGAATCGTTTGGGGTGATTATCTTGTTGCTGAGTACGATAGTTTTTAGAGTTTTGTTGAGATAATTTGTTACCTGCTAAGCCGGATCGAACCGGAGCGCTTGATCTCACTGGTGTGGAGCGTCTTCGGTGACTCTGATCCTTGGTTGTGAGTTCGTTTTAATCTTGAAGGTTGTGGTTTGTACCGCGCAAGACCACACCCGACGGGAGATCATTTAAACTTCTCATTTCTCTGGCAGCCCAGATTGGGGAGGAGGAGAAGTCAGTGGCCGAGAGTGGGATTGACTCACTGATTTTCCAGTCCCTCTTAGAACTCCTTCTCTCTTTCTACCGCAGGTGCTGTAGCGTTTCTTGCTCGTTGCTGTGCTCACTCATCAACTCTTCAAATGTGCTCTCTCTCCACAATGTCAACCAATACCCGGACTTGAAAGTTTGTTGGCGGACAACCATCCTATTCAATCATTTCAACCGACTAACCATCCATCCATCCTTCCGTCGTGGTATGTTTCCAAGGGGAATCGTGGATTGTTACCCTTCCGCGAGTAGCATCATCATCTTCGGCATCAACTGATATCGAATCAGGGACTTCGTTTTCGTCGTCCGCAGTGATCACTCTCTTCTGTCAATCTTCCTATCAGCGCACCACTAGCTCTTGCCGCTTCCCTGTTGATTCTCAGTCTGCCCCCGCCGGTGGAACAAGAGTCCCAGGCCAGCCAGCCAGCAACGGCTTCATAGCTAGTGGAAATCTCCTTCGGTTCATTTCGTTCATCGGAACCGCATCAATCGGTAAGCCCTCATTCAGTCTGTCGTTTGCATCTCGACAGCAGATCTCCTTTGCGCACTCAGTGTCGGTACTGGGCACTAACTTTGGATTCGCAGGGTTCCTGAACCCTGCTTGTAACGCTTTATCTCTCAGTTAGCCATGTGTACTGCCTACTTGCACTTGCTACCCCTAATCGCTAGCGTCAGAACCTGAATGCTTGGATTGTCATCTTGTCCGCCTGCTTGTGTCGGTGCTGCAAAAGGAGGGTCATTCAGGTTGAGTGGGCGCTAAACTTATTTCTGGCTGTTCATTCATCATCTCGCAGNGACTTACTTCAGGGTTGGTAGTGCACTGATTGACCAGTAGCAACTGATTTGGTTGCGAACGATGTGCATCACTCTCTTCTGCCTGCCTGTTCAGCATGTCATGGACTTCTCAGGGTTTGTAGTCCATTCAACAGATGGTGTGCATCACTCACGTAGACTGTCTATTTGTCGTCTGCCATGCTAGCTTATCAGTTTTTCTATTCCTTTCAGCCAGTGCAATTTAAGGTTGACTGAAACTGACTTCCGAGTGGCTGATATGCAGTGGTGCTGATTCGCCCATCGTCAGCAAGCTCGTGAGGCTCAATCTGGCAAGGTAACGCTCCAGCAATGGCGCTCTTCGATTTCGGTTTGTGGACTCAGCGCCCTCCTTGGCAGGAGTTTTGGGAGTGGGTTTGCGTTTCCTCTGCCCGTGCTGGCACTACCGTTGTAACTGCCCCGCGCTAGATGGCGAGTGGACGTTATCCTTCCTGAGCTGAGAACACAAGCAAGGGTGGGTGAGGGCGTTCATGGCGTGCCGGGCGACGCCTTGTTAACAAGGTGTTAAGCACCAATGGGTGTCCCGGCAGCCTTAGACGTCATCTATGGCTTCGTAGGGTGTGATGAGTGCTTTACCCGGCCCTCATCTTCTACGCAGCCCACCTACCCTTGTGACACGGGCACAGAGCTTCCTGGTGCAGCCCGTGTCAATTGTATATCACTCCTGAGCTAAGTGGATGAGAAGGAAGCACATGCGCCAATATGTTGGTGTTCAACTGCTATACTCTCTTCTAGTTGCAACGTAGCCTACTACTTGTTAACGTGCCTCCACTTGATGAAATAAAAGAGTAATTCGCCCTTGTTGAGACACA**

***Pseudotaxiphyllum elegans* (accession** [**QKQO-2009669**](http://www.bioinfodata.org/Blast4OneKP/search?Sequence=QKQO-2009669)**)**

**CTGGCTGCCCCCTTTACTATTAAACTGCCATCGTCTTGCCGGAGTTCCCACACTTGAAACGACTACGGCTTTGGAGCATGGTCCTCCTCCTCATCAAGGCCTTCAAGTGAGGTTCAGATTGCTTCTCTGGCTTCTGTCTGTAGCTGGCTCGATCACTATGGCGATGAGGCATCGCATCGCTCTCCGGGCTGTGTTCTCACAAGTGTCGTGGATAGCTAGTCTCTTGGGATCCTAGCGTTCAGTTGAGAGGCTTCCAGTGGTTTTGATTTCTGTAGATGTTTGTGTTTTTAGCGGTAGCAGTTGGTGTGCTTTTGTTTTGGGAATTCAGTTTGGGTTGGTGAAGAGTGAGAGTGATCAGTTGGTAGGGTTGAGACTACGCGAAGGTTCGACTGACTTTACAGCATTGGCAGCATTTGATTGGGGTTCCAAGGTGGAGCCTTTTGGGGTTGCTGTTGTTGTTGAGTACGCTAGTTATTAGAGTTTTGTTGTGGTATTATTCTGCTCATTGGGATCGGGAGCGCTCGATCTCACTGTGTGGAGCGTGTTCAGTGACTCTGATCCTTGTTGTAAGTTCGTTTCTGTTTTGGAGGTTGTGGTTTGTGTCGCGCGTGATCACACCCGACTTCAAGTGGAGATTGTTTAATCTTCTAAGTTTTCTGGCAGTCCAGATTGGTGAGGAGGAGAAATCAGTGGCCGAAAGTGTCATTGTCTGATTTTCCAATCTCTCTCTCTATCTACCCCGGGGTTTGTAGCGTTTCCCCTCCGCAGCTGTGCTCACTCAACTACTCTTCAAATGTGCTCTCTCTCCACAATGTCAACCAACACCCGGACTTGAAAGTTGGCTGCCAAACTACCATCCTACGAACCAACCATCCATCCATCTATCCTTCCATCGTGGTATGTTTCCAAGGGGAAGCGTGGATTGTTACGCTTCCGCAAGCAGCAGCGTCATCGGCATCGACTGATATCAAATCAGGGACTTCGTTTACGTTGTCCACAGTGGTCACTCTCTTCTGTCAATCTTCCAATCTGCGCGACGTTAGCTCCAGCCGCTTCCCTGTCGATTCTCAATCTGCCCCAGTGGAACGAGACCTCCAGGCCAGCCAGCTAGTGAGCAACGGCTCCGCAGCTAGTGAAAATCTTCTTCGGCTCATTTCCTCCATCGGAACCGCCTTTACTCGTAAGTCCTCGCATACAAAGCTCTGTCGTTTGAATCCCCCTGGCAATTCTTCTTCCTTGCCTCTCAGTGTCGGTACCAGCTTCGGATTCGCAGGGTTCCTGAACCCCGATTGCAACGCTTAATTTCTCAGATAACCGTGGGTACTACCTGTCTCTACTTGCTGTCCCCAATCGCTAGCGTCACAAAACCATAGAATGCTTAGATTGTCAGTAGATTGCTAACTCGTCCGCCTGCTTGTGTCGGTGCTGCAGAACCGAGAGTCCAGTGAGGCNAACCGAGAGTCCAGTGAGGCTGAGTGCGCGCTAAAGTATCTGTCACTTCTGGCTGTCTATTCATCATTTCACAGACTATATCAGGTCTGGTAGTACATCCAGCAGCAGCAAGGTCGGCAGAAACGGACTTCCGTGTGAGCGGTGTGCATCACGCACTTCTGTCTGTCTATTCATCATCTCGTAGATTTTTCAGGGTTTGCAGCCCATTCAGCAGTACTTACCAAGGTCGGTTGAAACTGATTATGTGCCTCAGTCACGTCGACTGTCAGTCTATTCTTCATCTTCCAGACTCATCAGGTTTTCGTAGTCCATTCAGCAGTGCTTACTAAAGGCGACTGGAACTGATGGTGTGCATCATTACTCATGTAGACTGTCTGTCTATTCATCATCTTCCAGACTTATCAGGTTTTGTAGTCCATTCAACAATGCTTACCTAGGTTGGCTGAGACTGGCTCCCTTCTGACTAGTGTGCAGTGGTGGTGATCCGGCTATAGACACCAAGCTCGTTGCAAGGGGAGGGAGGCTCAATCTGGCACGTTAGGTTCCAGTAATGGCAATCTCCAATCTCGGCTTGTGGATTCGTTGCTCCCCATGGCAGCAGTTTGGGGAATGGGTGTGTGTTTTTCCAGCGCGTGCTGGCATCGTCGTCGCTACTGCGGCGCTCTAGATGTCGAGCGGGCGTTATCCTTCCTGAGCTGAGAACGAAGGCAAGGGTAGGGGGAGGGCGTGCGTGGCGTGCCGGGCGACGCCTTGTTAACGGGGTGTTAAGCACCAACGGGCGCCGCGGCAGCTTCAGACGTCAGCTACGGCTCCGTAGGGTGTGATGAGTGCTTTACCCGGCGCTCATCCTCTACGCAGCCCACCTACCCTTGTGACACGGGCGCAGCGCTTCCCAGCGCAGCCCGTGTCAATTGTATATCACTCCTGAGCTAAGTGGATGGGAAGGAAGCACATGCACCAACAAGGCGTTGTTGGAGTTAGTTGTAGTACTACTCTCTCTCTTCTGGTTTGCAATCTAGCCTACTAGTTGTGTGAACAGGCCTCCACTTGATGAAATAAAAGAGTAATTCGTCCCTGGTGGAACTTAATTTTGGGTCCCTCA**

***Philonotis fontana* (accession** [**ORKS-2058791**](http://www.bioinfodata.org/Blast4OneKP/search?Sequence=ORKS-2058791)**)**

**GCAAAGTTATTTAGCTGAGATAAGATTGCTACGGGAAGTCAAACCTGGCCACGGACGATAAGTTAACTTGTTACCTGGCTGGCTGGCTGGCTCCTTCATTGCCATTCACTATTAAACTGCCATCGTCTCCATCCTCCTTACACACTTTGAACTACGGCTTGTGAGTTCTTCACGAAGCCAGTGAGAGCTCCACTAGTCTCTTCCCTCCCCTACCAGAGTTGGTTCGTCGATCGCCAGTTGAACTCCATGGTAACCGTGAATGGCCTGATCGGCCGCATCACACTCTGGGCGGTGTTCTCCGGAGTGTGGTGGACAGCTAGTCTACTGGGATCCTAGCTTTGACACGGTTCAGCAGTGCTTTACTTTGTAGAGGTCGACGAGTTGTTTAGTGATACTTTACGGTCTGCGTTGATAGCGCGTGTTGTTGCGTTTTAAAAAACAATTCTGTGAATTGTTTTGTTTTAGTGAGCGGTGGGAGTGATCAGTTGTTGGTAGGGTTAAGTACGTTCTTGTGGGAGTAGGAAAGGTTTTTCAACAGTTCGCTGCATATGATCAGTGTTTGCCGCATTTGATCAAGTTGTTTAGTCCAAGGGTGCAGCTTTTGTTGAGAGCTAGTTTCAAAAGTAGGAGAGTTTTCATTGTTGAGAGTTTTGCGGTGACATTTTTCCCCTCTTTGGGATCGGTAGTGTTGTTGTTGTTGTTGTTGTGGTGTGGAGTTTGGTACTGATCCTCGCTGTGAGCCCAAGTTTTTTTTTGTTTTGGTTTTTGGCTCACGTGATCACACGGTCGCTACTTGGAGACCGGGTTTGCTTCCAGGAGATTTAATATTTAATCTTCTGTGCTAGCTATCGGCCGCTGCCCACAAAGAGAGAGAGAGAGAGAACAGTGGCATTGACTGATAATTTTCAATCCCTGCCAGGTTGGCTGATAGTTTTGCAACCGTTCCCTTTTGCCTCACAGCTGTGCTCACTCATCAACTCTTCAAATGTGCTCTCTCTCCACAATGTCAACCAATACCCAGACTTGAAGGCCGAGTTTGCTAACGAAGTACCTCGGTGTATGTTCCGTGTTGATTGTAAGCTGCTGCATCGGCATCTGCTCCAGAATATTATTCCAGCCACGTGAATGAGAATCAGGGACTGTGTCGTCGTCCACAGTGGTCACTCTCTTCTGTCAAGCTTCAACCCAGCTGCCGGTTCAACCGCTTCAGGCTTATTTATCAATCCACCCAACCCGGTGGAGCGAGAACTCGAGCAAGCGACGGCTCAGCGAGAAGCTACACCCGCTCACTTCTTCCTCGTAACCGCCACAGTCGTGGTTTTCACGTCAACGTGGCTGGAACCACCCGGCATTGACACACTTCTTTCTGCAGTGGAATTTCCGGGTCTCCGAGGATCTGTAGTGGGAATTTAATCCCGTAAGGTAACCAACCAGCTCCCACCGGAGCTGCTGCTGCTGCTACAATGGTGCACGCCGATCTCTGCATCTCGGCAGGGAAACCGTTCATCCGACTACCGCCAGCATCACAATACTACTATTACAACTTGTGTGTCCCAACCATAGCCCTGGAGCCAGTTCACTACTTCTACTGCTGCAGCAACTCTGCAACCGTGGTTTACTAGTGAATGAGTGGGCGTTATCCTTCCTGAGCTGAGAAAGAGAAGGGGAAGGGGGTGATCACCGGAGTCGAAGGGCGCCTTGTTAGCGGGGTGTTAAGCACTAATATGTGGCACTGCGCCTTAAGACGTCAGCTATGGCTCCGTAGGGTGTGATGAGTGCTTTACCTGGCGCTCATCCTCTGCCCAGCCCACCTACCCTTGTGACATGGGCCGTGCTCTCCCGAGCACGGCCCGTGTCAATTGTATATCACTCCTGAGCTAAGTGGGAAGACACCAAGACCTTAACACTATTATTATTACAGTGGGGTAGTAGTAGTTATTAGTGTCGAAATCGCCCAACTAGTGGTGTCTTTGTTGAAGTCACCAGTTTGCAACTGTGATGAGATGCTTCCACTTGATGAAATAAAAGATTAGTCCCTCGTGTAACCCAAAAAAA**

***Hedwigia ciliata* (accession** [**YWNF-2050742**](http://www.bioinfodata.org/Blast4OneKP/search?Sequence=YWNF-2050742)**)**

**GACGGTAGAAGATCGGATCCATGAATTTGGCTCTCTTTTTTTTTATCAAGTGAACGCATCGACACAATATACATCAGTGCCTTTCCACAGGTGGTGCGATCCCAAACTAGAACTAGTACTATTATTACTACCCCACAGGCCACAGCAATGCTACTCTTGGTCTTGATGTGTTGTTTCCCACTTAGCTCAGGAGTGATATACAGTTGACAAGGGCCGCACCGGGAAGAGGCCGGCCCATGTCACAAGGGTAGGTGGGCTGGGCAGAGGATGAGCACCAGGTAAAGCACTCATCACACCCTACAAAGTTGTAGCTAACGTCTTAAAGGGCAGTGTCGCACGCTAGTGCTTAACACCCCGCTAACAAGACGCCGGCCCGTTTCCACCCTCCTCACCCTTACTTTCTTTTCTCAGCTCAGGAAGGATAACGCCCACTCATTCACTAGTAACCCGCGGTGGTTGCAGGGATTTTGTGGCAACAACAAGCCACGGTTGCAGAGTTGCGGGTGTGAAGTGGAGGGACACACAAGGAGTAGTTGGAAGGGGGCAGTCGGATAAACGATTTCCCTGCCGAGATGCAGAGATCGGAGTGCACCATTTTGCTCCGGTTGAGTAGTTACCGAGCTGGGATGGACGATGGGGCTCAACAGCTTACACAATTCCTCAGAGCTGTGTCAATGCGGAGTGGCTCCAGTCGAGTTGACGTGACACCCACAGCTTAGGCGGTTCCGTGGAAGAAGTGAGCGGATGGTTGTAGCTTCTCGCTGAGCCGTTGCTTGCTGGACTTGTCGCTCCACAGTAAACTGCTGGGTGGATCGATAATCGACAGGAAAAGCGGTTCAACCGGAAAACAGACAGACGGAAGCTTTGACAGAAGAGAGTGATCACTGTGGACGTTGACGACGACGACGACACAATCTGTCATTCACGTGGGGAGGGGCTTGAATAAGTCGGGCCAGATGCTGATGCTCTCATAAAGCTCACAACCAACGGCGGCTCGGTGGCGAACTCGGTGCCCTTCAACAAGTCTGCGTGTTGTTTGACATTGTGGAGAGAGAGCACATTTGAAGAGTTGATGAGTGAGCACAGCTTTGGGGGAAATCAGTCAATGACGACTCAATGCCACTGATTCACCGATGATCTCTTTCCTCGGCCAGTCTGGACACAGCGCTGCTGGTAGCGGCATAGAAGTTTGAACAAATCTCCAAAAAACTTGCGCTCACAGGGCGAGGATCAGCGTCTCCGCAAGGATCAGCAAGTAAAGCTCCACACAGAGAGCACTGGTTACCGACCCCAAACGAGGAAAATATCACAACAAAAACTCGCAACTTGGAAGAACTCAAACCACTTGAGAATCGAAAACTAGCTCAGTGACCAGGAAAGCTGCAGGCACCTTCTGCAGGCACCAGATGCGGGCAACCCCTGATCCAACTGCAGCAAGCAGAAAAACACAAGGAACTCAACCCTACCAAACGGACCACTCTCACCCTTGACTAAAACCAAAACAAAACAAAAAAAACACCACCTCTACAAAAAGCCACAGCTAGGATCCCAGCAGACTAGCTATCCAGCCCACTTTGGAGAACACAGCCCAGAGTGCGGAGCAGCAGATCAGAGGGCTGTGGCATCGATTCGCCATCGCCACCTGCCGATCGAGCCCTCGCTCGCTCACACACACACAAACGCAGGTGTAAGACGAAGCTTGAAGAGAGAGCAGTGACGGACCCCTCCAGTGAACGGCCAACAAGCCGTTAAGACCCCAAGTGTGAGAAGAGCAGTGCAGACGATGGCAGTTTAATAGTGAACGGCAATGAAGGCGGGGAGCCAGGGTAACTTGGGCGTCCATCCGTCCAGGTTTGACTTACTTCCCCCGCTCGATCGCAATCGCTACTACTCCGGTACTCGGGACCCTCGGTAGCTT**

***Schwetschkeopsis fabronia* (accession** [**IGUH-2166854**](http://www.bioinfodata.org/Blast4OneKP/search?Sequence=IGUH-2166854)**)**

**AGGTTGAAAATAACCGCCCATGTTATAAGTTACTGCTTTCGGACTCCCCTCGAAGTCAAACCTCGACGGACCTGGCTGCCCCCTTCACTATTAAACTGCCATCGTCTGGCTGGAGTTCTCACACTTGAAACGACTACGGCTTGGGAGTGTGGTCCTGCTCCTCCTCAAGGCCTTCTACTGAGGTTCAGTTTGCTTCTCTAGCTTCTGTCTGTGTCTGCCTCGACCCCTATGGCGATGGGGCATCGCATCGCTCTCTGGGCCGTGTTCTCGCAAGTGTCGTGGGTAGCAAGTCTCTTGGGATCCTAGCTTTTAGTCGGGGAGGCTTCGAGTGGTTTCGCTTTCTGTAGAAGTCTGTGTTTGTAGTGGTAGCAGTTGGTGTGTTTTTGTTTTGGGAATTTAGTTTGGGTTAGTGAAAAGTGAGAGTGATCAGTTGGTAGGGTTGAGACTCCGCGAGTGTTCGACTGACTTTACTGCATTTGATCAGGGTTTGCCGCATTTGTTTGGGGTTCCAAAGTGGGGCCTTTTGGGGGTGGTTGTTGCTGCTGAGTACGTTAGTTTTTAGAGTTTTGGTGGGATAGTATTCTGCTCACTGGGATCGGGAGTGCACGATCCCATTGTGTGGAGCGTGTTCGGCGACTCTGATCCTTGTGAGATCGTTTTTATTTTGGAGGTTGTGGTTTGTGTCGCGCGTGATCACACCCGACTTCAAGGGGAGACGGCTCAATTTTCTGATTTTTCTGGCAGCCCAGATTGGTGAGGAGGAGAAATCAGTGGCTGAGAGTGTGATTGACTGATTTTCTAATCTCTCTCTTTCTCTACCCCGGGGTTTGTAGCGTTTCCCCCTCGCAGCTGTGCTCACTCATCAACTCTTCAAATGTGCTCTCTCTCCACAATGTCAACCGACACCCGGACTTGAAAGTTTGTTGGCGAACAACCATCCAATAAACCAACCATTCATCCATCCATCCATCCATCTATCCTTCCATCGTGGTATGTTTCCAAGGGGAAGCGCGGATTGTTACGCTTCCGCAAGCAGCAGCATCATCGGCATCGACTGATATCAAATCAGGACTTCGTTTTCGTCGTCCACAGTGGTCACTCTCTTCTGTCAATCTTCCAATCTGCGCGCTGCTAGGTCCAGCCGCTTCCCTGTTGATTCTCAATCTGCCCCAGTGGAACGAGACCTCCAGGCCAGCCAGTCAGCCAGCAACGGCTCCGCAGCTAGTGAGAATCTTCTTCGGCTCATTTCCTCCGTCGGAACCGCCTCAACTGTGGTGGTGATCCGGCCAACGACACCAAGCTCGTTGCAAGGGGAGTGAGGCTCAATCTGGCAAGTTAGGCTTCAGTGATGGCACCCTCCAATTTTGGCTTGTGGATTCGGCACCCCCCTTGGCAGGAGTTTTGGGAGTGGGTGGGTGTCTCCCACGTCCGTGCTGGCACCTTCGTCGCGACTGCGGCGCGCTAGATGCCGAGCGGGCGTTATCCTTCCTGAGCTGAGATTGAAGGCAAGGGTGGGGGAGGGCGTGCGTGGCGTGTCGGGCGGCGCCTTGTTAACGGGGTGTTAAGCACCAACGGGCGCCCCGGCAGCCTCAGACGTCAGCTATGGCTCCGTAGGGTGTGATGAGTGCTTTACCCGGCACTCATCCTCTACGCAGCCCACCTACCCTTGTGACACGGGCGCAGCGCTTCCCGGCACAGCCCGTGTCAATTGTATATCACTCCTGAGCTAAGTGGATGGGAAGGAAGCGCATGCACCAACGAGGCGTTGTCTGAGTTAGTTGTAGGACTACTCTCTTTTCTAGTTTGCAATCTAGCCTACTAGTTGTGTGAACATGCCTTCACTTGATGAAATAAAAGAGTAATTCGTCCCTGGTGGAAATCAAAAAAAAAAAAAAAAA**
