# Supplementary material for: TAS3 miR390-dependent loci in non-vascular land plants: towards a comprehensive reconstruction of the gene evolutionary history
Source: PeerJ. 2018 Apr 16;6:e4636. doi: 10.7717/peerj.4636 (PMC5907777; doi:10.7717/peerj.4636)
Supplement: Figure S5 [file peerj-06-4636-s005.doc]

**Figure S5.**

**Conserved portions of SGS3 amino acid sequences used for constructing of phylogenetic tree.**

Sequences of vascular plants are in green, and sequences of non-vascular plants are in blue. Positions of amino acids conserved in zf-XS domain are in yellow, and positions typical for RNA recognition XS domain are in brown. Accession numbers are shown above the protein sequence.

***Sphagnum fallax***

**Sphfalx0029s0100** (PHYTOZOME)

SDMTQKSHETQKKLKWFRDFFQDLDCLTDAQLHEHDRQWHCPPCKGGVGGTEWFRGLGPLATHARTMRSRRVKLHRNFAEVLDEELRLRRVGANTSGPESMFGKWKGLREDDEAQFSLIVWPPMVVVENTRLEKDEQEKWTGMGNKELMEYFKEYNPVKPRHAYGPQGHRGMSLLIFGNSPAGYHDAEHLDKHFKDSRRGRDDWNRPSKLLFHPGGNRILYGYLAVREDLETFNRHSRGKTVVKYEMKSLREAVLEPMQRIGEQTQHMQQLQNKVEKEQEVSKVLKETVSMVSKNLSLREQELQILRRRAEEQHARNKQEQAEMEEFFQAERDQLAEEFNMKEAELEKLRD

***Marchantia polymorpha***

**Mapoly0033s0105** (PHYTOZOME)

ESHKSHATQRRNKWFRNFFQDLDELTEDELQEHDRQWHCPACQGGVGAIDWYRGLQPLLAHAKTVRSKRVKLHKAFAEVLEEEVRIRGAAPGTHGSGKYGKWRGLGDDNDNKDTLIVWPPMVVIRNTQLEQDEVDKWIGMGNKELLDLFKKHNPVKARHAYGPQGHRGISILIFPESPTGYYDSQRLDKHFRDALRGRDHWYSQGKVLFQPGGDRILYGYMATAEDLDVFNKHSKGKQNLKWEKKSLQEMVLIPMRRIDEENNKVVYLQGQVQREMEVGKTLKKTMSVLMKKLRMREDELKVIRERARDQYKQQEMEMGDMETLYKSKVEQLKQEIMEKEAGLQKVYE

# *Physcomitrella patens*

**XP_001773905**

FAQESHEDQKKIKWFRDFFQDLDSMTDAQLNEHDRQWHCPACKGGPGAIDWFRGLVPLATHARTMRSRRVKLHRKFAEILEEELRIRRSAQPNVELQSTFGKWNGLSEDTESQQKMIIWPPILVIQNTQLEQDDEGKWIGMGNKELVDMFKDYNPLKPRHAYGPQGHRGMSLLIFPDSPIGYHDAVRLATHFANSRRGRDDWQRPGKILFKPGGERILYGYLAIK

DDLDIFNRHSRGKTLLKYELRKLQQVVLEPMQRMEQQNQLIQSLQIEQNTLKKSVSETSKNLQLREEELQILWDRQGKQHAENKRELADMEEFFQQESAQWANEVAAKDSELQKLRD

***Anthoceros punctatus***

**ERX2100252**

EDSDSEAEDFQANSKWFQPFFDALRNLSVEQMSEFDRQWHCPACKGGVGAIDWYRGLQPLLTHAQTIRAKRVKLHRMFASLLEDELRQRGAAASAAVAGKFGKWKGLRETDESLNPLVLWPPMVVVQNTQLEMDEQDKWIGIGNQELLDHFREYNAVKARHAYGPQGHRGMSIVIFGDSPSGYYDAQRLDKAFKEARRGRDDWYRQSKTLYQPGSSGQRILYGYMANKEDVEVFNRHGRGKMLLKCEMKRLNETVLEPMRQQDEDFHQVTYLKDKVQKAQQHRKELERTVCVITRQLRLREEEIKEIQMRAEEQNVKNKKSLEDMEKYFEEMLQTYRDKVALEEDKEQEKLREA

[***Takakia lepidozioides***](https://www.ncbi.nlm.nih.gov/Taxonomy/Browser/wwwtax.cgi?mode=Info&id=37425)

**ERX2100030**

ATQRSHETLKKLKWFRDFFQDLDNLSKEQLHGHDRQWHCPACKGGVGAIDWFKGLGPLLAHAQTMRSRRVKLHCKFAEVLEEELRLRRAGPGSEWDALFGKWKGLHDDTDAQDALIVWPPMVVIQNTQLELDENETWLGMGNKELLDYFQEYNPLKPRHAYGPQGHRGMSLFVFGESPSGYHDAQRLNKHFKDAKRGRDDWDSPGKLLFHPGSGGSRILYGYMADKEDIEIFNRHCKGKTVLKYELKKLQEMVVKPMRRMDGQSQQVLYLQTKVEREMEVSRTLKKTVSVVAHNLQLREQELHIMRRRAEDQHLRNKLQQADLEEFFKIERNQLADELEAFQAESAQWADE

[***Barbilophozia barbata***](https://www.ncbi.nlm.nih.gov/Taxonomy/Browser/wwwtax.cgi?mode=Info&id=209798)

[**ERX2100042**](https://www.ncbi.nlm.nih.gov/sra/ERX2100042%5Baccn%5D)

DSHKSHSTQRMNKWFRNFFQDLDVLTEEQLHEHDRQWHCPACQGGVGAIDWYRGLQPLLAHAKTVRSKRVKLHKSFAEVLEEEIRIRGAAPGTHGSGKYGKWRGLGDENESKHIVWPPMVVVRNTQLEQDEDDKWIGMGNKELLDLFKKHNPVKARHAYGPQGHRGISILIFAESPTGYYNAQRLETQFNEALRGRDHWYAAGKLLFQPGGDRILYGYMATPDDLEIFNKHSKGKQNLKWELKSFQDMVLSPMRKLDEENTKVTILQGKYQREQETSRALKKTMSQISKKLEMREEEVQVIRKRAKEQQQHYEIELKDMEDLYKEKRLFRPLEEAFQAESAQWADE

***Pohlia nutans***

**GACA01004697**

YTQESHEDQKKIKWFRDFFQDLDGMTEQQCNEHDRQWHCPACKGGPGAIDWFRGLAPLATHARTMRSRRVKLHRKFAEVLEEELRLRWSGAPCTGVVSVDGTQLAFGKWKGLSEETEAQQQQLVIWPPIVVIQNTQLEQDDEGKWIGMGNKELVDMFKDYNPLKPRHAYGPLGHRGMSLLIFSDSPAGYHDAVRLGNHFVNLRRGRDDWKRPSKLIFRPGGNRILYGYLAVKEDLDIFNRHSKGKTLVKYDVRRLHEVVVEPMQRMEQENQQMQSLQSEHNTLKKAVSETSRNLQLREEELQVLRDRGEKQHAENKRQLADMEEFFQQESAQWADEVASKDTELQKLRD

***Klebsormidium nitens***

**GBSO01024386**

ELEGFEGLAKEKEEKAYKKYRRVYLELLQEASKGDGAKDQWKCLVCKNRTKFWFNSLHALHQHAETKKNEWRIQHRGLARALRELLSRDPMGPLGILLPPVEDDDQIIVWPPAVILQNTRVRMDADGHWNGLGATEIRDQYKSFNPMAVRPAYGPDGHKGCVVVQFAADPYGFDKAVQLSQYFEKQSRGREGWESHGIRSKRFDEEGNLLVFGYLAQPKDMWQLDKFAKGKNGKAGQLVKWWTESKKEAVDDEKARREQQRLAEAGEVSRLREKAAEDEGARARAESELRVQSKKLRNLQERYAKMEQLQQQQKDKHEEELESQELFYDGKFKELATQFTATEQDALAKRR

***Nitella mirabilis***

**JV762822**

GGDERLIPRVELERYSKKYYGKFIAKLNAADVGDWEAINEHCPVCKTVEFFTSLSALALHAKTKKSKTKAHRAFAHAIIKLKETKAKENASDIPSSSQIWRSMEASSDVQIVWPPMVMVMNTRKTKDPTNGKWIGMGSNELRPLLPAHPYMRDKHSYGPDGHRGISIVEFESSPAGYMMANDLHDDFRKQGKGREAWKSARKVLMGTENKQGLQKNLFGYLATEMDLMEFNRHCTGKKSKLGMKYRMVSRLEMVVEPLKLSEQENKELKLCRSQAALATQNMAALERQRQEELQRYHRKSAELDVQKEMMKKLEEEHNRERKERERFYEKRLREGREEQEKRMQELKTKLI

***Coleochaete orbicularis***

**GBSL01051614**

DDSASDEGSDDEHLRYEKLYARFCKCAEDWSMDDINDGFFHCPVCRGKPGAGTWYRGLRHLIQHAKDYKKHRFDAHKEFADVMRTWMEKYQVSSGGLFGSAPRERWVGPDDEEVEEVESRDIVWPPMVLVRNTDLYLDDDKWVGMGNQELRDKFKKFNMVNSEHAYGPQGHRGMSMLVFERTPAGWMEADALARKFAQNGTGRMEWERLPRRKVFRDPEAKHILYGYLADAGDVEYFNQHRKEKDKLKFSLRKYGDAVVRPMRKAEEERAELQKSHLRALAAIEEKEVLSKENKELMGKI

LSRDKQLTIHVKLIDDLQTKHKEEIKSLEEFYKEEVDEISEKWSKDKDIVTKKKE

***Spirogyra pratensis***

**GBSM01015566**

EDEDEEVKKKRIKCLKHMEAFLTELVSYSNPELSDNSREWKCKACVGISGEISWYPGISALVAHCNTLKKKNVKEHRYLAKSIKSILEKKGIAGSVCGGKVQGHWKGLEKVNEQEKCIVWPPMLVIRNTRLFMDSEDKWVGMGNKELVESFTSCKNIKRGRHAYGPQGHRDLSLLIFNSSPVAYHEAVALDKELSANARGRKEWERPSQPRVGQDNKKLLYGYLAEPHDIVEFNRHHKGNQVVDFKLKKMDELATELQKLQEEVVKVTKLSFKVEQLSQEKQELKMKYDMLENSASLHNE

QESKLKRKNGEIEALHKITEDNKKAHEKEVISMEQHYQMKIQEMNQNFTKSLSEIQKQHH

***Azolla filiculoides***

[**GBTV01027099**](https://www.ncbi.nlm.nih.gov/Traces/sra/sra.cgi?run=<@run@>.<@spotid@>.<@readindex@>&RID=3UM91TP6014&display=reads)

IPMQVSKVLIRENRRNVFAVFFQTLDEFSNEQILEHEREWHCPACQCGVGAIQFYRGLQPLIDHAKTRQTRASLHRKFAEVLEEELEVRRLGTRGSETVFGKWRGLQHDDASQDQNVVWPPIVVIQNTQLHKDDQEKWIGMGNKELLDLFQDYSPTKARHSYGPQGHRGMSVVIFAESPTGYFNADRLATKFRNSQRGKHNWENPGKRIFEPGGDRILYGYMATAEDMEIFNRHCSGKLKLKWEIKKYQAAVVEPMNKMNEQNQRLHYYKAKTQKQEQLSKTLERSVSKFTKALEMREIEIAELRKKYKEQDEQKEREMDDMEQTYRERIKQLQTKVARSEQEEQEEQE

***Lygodium japonicum***

[**FX957669**](https://www.ncbi.nlm.nih.gov/Traces/sra/sra.cgi?run=<@run@>.<@spotid@>.<@readindex@>&RID=3UM91TP6014&display=reads)

DSGGSAVSHETAKKDKQLRQFFDTLSGLTVQQLNEYDREWHCPACQGGVGAIDWYKGLQALLAHANTKRSKRVKLHRKFARILEEEMRRLGTSAGENELMFGKWKGLRQDNEIVNPLIVWPPAVIIQNTRLELDDQEKWTGMGNKELLDYFKDYNVLKAKAAYGPQGHRGMSILVFPETPTGYMDAQRLDKCFKDEMRGRANWDKPGKVIFYPGGKRILYGYMASSEDMDIFNKHAGKSKLKWSLRRLSEVVVVPMRQMHEDNEQLNHLKVKVEQQKEQSKTLEKTVSMITNTLRLREEEIQVIRHQALEQHNQNQAEMDELEKVYKNKLNQLMKRRAKQEEELQQKE

***Acrostichum speciosum***

[**GEEJ01008259**](https://www.ncbi.nlm.nih.gov/Traces/sra/sra.cgi?run=<@run@>.<@spotid@>.<@readindex@>&RID=3UM91TP6014&display=reads)

DDSDASVKSLETRKKNKWFHSFFDTLDEFTNEQIMEHDRQWHCPACHGGVGAIDWYRGLQPLLAHAKTHRTKRIRLHREFAKVLEEELDMRRAGTSASGETKFGKWKGLRNADATMDEMIIWPPMVVIQNTQLTQDEQDKWTGMGNKELLDMFKHYSPVRSRHSYGPQGHRGMSVLIFAESPTGYYHAERLAKAFKDAGKSRDQWDVPGKRVFQSGGDRILYGYMANAEDMDIFNRHHAGKSKVKWELKRYKEAVAEPLSQMDKDNRQLHYLKVKMQQQKEQSRILKKSMSMFSRKLQQREEEIAIIRQLARDQYEEYQREMDDLERTYKERSMQLQKNILKREQEVWEKKE

***Ceratopteris richardii***

[**GBGN01029655**](https://www.ncbi.nlm.nih.gov/Traces/sra/sra.cgi?run=<@run@>.<@spotid@>.<@readindex@>&RID=3UM91TP6014&display=reads)

EDDDSDASIKSLESRKRSKWFRSFFNELDAITNEEITDHNRQFHCPACQGGVGAIDWYKGVHPLLTHAKTHRTKRIRLHREFAKTLEEELEMRTVEIASLGGTRFGKWRGLQNTDSTKDMMIIWPPMVVIQNTQLTRDEHDKWIGMGNKELMEMFQDYTPAKARHAYGPQGHRGMSLLIFPESPTGYWYADRLAKVFNDAGKGRQHWDSPGKRVFQPGGDRILYGYMARAEDLDIFNKHSAAKSKIKWTLKRYREAVDKALSQMDEENQQLIYLKSKVQKQKEQSKILEKSLGTFSRKLRQKEEEIFKIRQLARDQHEENQREIDELEKTYKERIIQLQRDRLKREQQIQEKKE

***Cycas revoluta***

[**GBJU01014166**](https://www.ncbi.nlm.nih.gov/Traces/sra/sra.cgi?run=<@run@>.<@spotid@>.<@readindex@>&RID=3UMHHCKM014&display=reads)

DDGWDSIAVSHEALKNNKWFKGFFEQFDGLTLKVIDETQWQCPACQGGGAKGAGSFKGMQPIITHAKTIRSKRVKLHRKLAEVLEEELRRRGAACLVAEEMFGKWKGLREPVNNQEIVWPPIVIIQNTVLDQDENEQWIGMGNKELLEYFKGYKAAKARHAYGPKGHRGMSVLIFEDSAMGYLEAERLHKHFLKEGRGKDDWERRRILFHPGGKRVLYGYLATKEEMEIFNRHSKGKAKLKYDMRSYHSMVVEPMRQMNEDNQNLIWLKTKVAKEQEHSRTLEETVSIVARKLRMREDEIKIIRQRATEQHEECKKEMDYLELSYRQQIDQLYDDVARREQELEKMHE

***Ephedra trifurca***

[**GBKT01024311**](https://www.ncbi.nlm.nih.gov/Traces/sra/sra.cgi?run=<@run@>.<@spotid@>.<@readindex@>&RID=3UMMX0K1014&display=reads)

SNMSHETKKKNKWLRSFFESLDNLTFEEMTDPQRQWHCPACAGGVGAIDWYRGLQPVLAHAQSIRTKRIKLHRCLSSLLNEELQRRGAGCSIIEEKFGKWKGLGETNSDTEIVWPPMVIVQNTLLEKDDNEKWLGMGNKELMEYFKDFKAIKARHSYGPKGHRGISALIFEDSAIGYMEAERLHKEFHRKGRGRAAWEVPYPTLHQPGGQRILYGFYAKKEDIDTFNIHSKGQTRLKYDMKSYNEKVFEPMRQMDENNQKLHYLQTKVAKQEDYSRTLEKSFGEVNTKLRMKEKEIETIRQRTSEQHEQYIQEMDFLEKIYRKQIADQKLHLDDREQELEKMHK

***Ginkgo biloba***

[**GBYR01009869**](https://www.ncbi.nlm.nih.gov/Traces/sra/sra.cgi?run=<@run@>.<@spotid@>.<@readindex@>&RID=3UMPZNSK014&display=reads)

DSEASDVSHGTLKNNKWFKSFFQSLDGLTVDEINEPERQWHCPACKGGVGAIDWYRGMQPILAHAKTKRSKRVKLHRKLAELLEEELRRRGAACVIAEEMFGKWKGLRDTVNNQEIVWPPIVIIQNTILDQGDCEQWIGMGNKELLEYFKGYKAMKARHAYGPKGHRGMSVLIFEDSAMGYLEAERLHRHFLKEGRGKDDWERRRVLFHPGGKRVLYGYLATKEEMEIFNRHSKGKSKLRYDMRSYQSMVVEPMRQMDEDNQNLIGLKTKVAKVQEHSKTLEETVSIVARKLRMREDEIKIIRQRATEQHEECKKEMDCLEQSYRQQIDQLYEDVSRREQELEKMHE

***Araucaria cunninghamii***

[**GCKF01043049**](https://www.ncbi.nlm.nih.gov/Traces/sra/sra.cgi?run=<@run@>.<@spotid@>.<@readindex@>&RID=3UMZHMV2014&display=reads)

ESAGSDVSHETMKKNRWFKTFFNSLDSLTVDQLNEPERQWHCPACRGGVGAIDWYRGMQPILAHAKTMRSRRVKLHRKLADVLEEELRRRGAACVIAEEMFGQWKGLQESVVNQQIVWPPIVMIQNTLLDQDENELWIGMGNKELLEYFKGYKAMKARHAYGPRGHRGMSVLIFEESALGYMEAERLHKHFLKEGRGKEDWERPGKVLFHPGGKRVLYGYLATEEEMEIFNRHSKGKSRLKYEMRSYQQVVVESMKQMSEDNQKLTYYKNKVAEEQQNSKTLEETVSVVSTKLRMKEAEIKIIRDRATEQHEESKQEMDNLEQSYRQQIDQLNEDIVKREEELERMQD

***Cryptomeria japonica***

[**FX336376**](https://www.ncbi.nlm.nih.gov/Traces/sra/sra.cgi?run=<@run@>.<@spotid@>.<@readindex@>&RID=3UN2USJK014&display=reads)

ADVSHETKKKNKWFKSFFESLDSLSIEQINEPERQWHCPACQGGVGAIDWYRGMQPILTHAKTVRSKRVKLHRKLAEVLEEELNRRGAHAISEEMFGKWKGLRETVNDREIVWPPIVIIQNTILDQDENEQWIGMGNKELLEYFKGYKAMKARHAYGPKGHRGMSVLIFEESGMGYMEAERLHRHFLKEKRGKEEWERHKVLFHPGGQRILYGYLATKEEMEIFNRHSKGKQRLKYDMRSHHQMVVESMKQMNEDNQKLTFYKTKVAKEQEHSKTLEETVYQVSSKLRLREAEVKVIRQRATEQHEESKKEMDYLEQSYRHQIDQLNEAIAEREQELEKV

***Cephalotaxus hainanensis***

[**GBHQ01002898**](https://www.ncbi.nlm.nih.gov/Traces/sra/sra.cgi?run=<@run@>.<@spotid@>.<@readindex@>&RID=3UN7V7EN014&display=reads)

ADSDGDDVSHETMKKIKWFRSFFESLDGLTIEQINEPERQWHCPACQGGVGAIDWYRGMQPILTHAKTIRSKRVKLHRKLAEVLEEELRRRGAHAISEEMFGKWKGLRETVNDREIVWPPIVIIQNTVLDQDENEQWIGMGNKELLEYFKGYKAMKARHAYGPKGHRGMSVLIFEESGMGYMEAERLHRHFLKEGKGKEDWERRRVLFHPGGQRILYGYLATKEEMEIFNRHSKGKARLRYDMRSHHQMVVESMKQMNEDNQKLTFYKTKVAKEQEHSKILEETVSDVSSKLRMRDAEVKIIRQRATEQHEESKKEMDYLEQSYRQEIDQLNEDIAKREQLSEKMHE

***Pseudotsuga menziesii***

[**GFFY01006185**](https://www.ncbi.nlm.nih.gov/Traces/sra/sra.cgi?run=<@run@>.<@spotid@>.<@readindex@>&RID=3UNATFAU014&display=reads)

DSEGSDVSHDTLKKNKWFKLFFESLDSLSIDEMNDPERQWHCPACSGGVGAIDWYRGMQPIVAHAKSIRSKRVKLHRKLAELLEEELGRRGAACVIAEEMFGKWKGLRETVNDQEIVWPPMVIIQNTLLDQDENEKWIGMGNKELVEYFKGYKAMKARHAYGPRGHRGMSVLIFEESAMGYLEAERLQKQFLKEGRGKDDWERRQVLFYPGGKRILYGYLATKEEMEIFNRHSKGKARLKYDMKSYHKMVVEPMKQMDEDNQKLTWFKSKVAKQQEHSRTLEETVSKVASKLRMKDTEIKIIRQRATEQHEESKKEMDYLEQSYRQQIDQLNADITKRESELEGMQE

***Larix kaempferi***

[**JR169809**](https://www.ncbi.nlm.nih.gov/nucleotide/JR169809?report=genbank&log$=nuclalign&blast_rank=1&RID=3UNG3H96014) **and** [**JR149947**](https://www.ncbi.nlm.nih.gov/nucleotide/JR149947?report=genbank&log$=nuclalign&blast_rank=3&RID=3UNG3H96014)

DSEGSDVSHETLKKNKWFKIFFESLDSLSIDEMNDPERQWHCPACKGGVGAIDWYRGMQPIVAHAKSIRSKRVKLHRKLAELLEEELGRRGAACVISEEMFGKWKGLRETVNDQEIVWPPMVIIQNTVLDQDENEQWIGMGNKELLEYFKGYKAMKARHAYGPKGHRGMSILIFEESAMGYLEAERLQKQFLKEGRGKDDWERRRVLFYPGGKRVLYGYLATKEEMEIFNRHSKGKARLKYDMRSYHKMVVEPMKQMDEDNQKLTWFKTKVAKQQEHSRTLEETVGIVASKLRMRDAEIKIIRQRATEQHEECKKEMDYLEQSYRQQIDQLNADVTKRESELEGMQE

***Abies pinsapo***

[**GCZN01009339**](https://www.ncbi.nlm.nih.gov/Traces/sra/sra.cgi?run=<@run@>.<@spotid@>.<@readindex@>&RID=3UY7JBFF015&display=reads)

SDVSHETRKKNKWFKDFFESLDSLSIDQMNDPERQWHCPACRGGVGAIDWYRGMQPIVAHAKTIRSKRVKLHRKLAEVLEEELGRRGAACIIAEEMFGKWKGLRETVNDQEIVWPPMVIIQNTLLDQDENEQWIGMGNKELLEYFKGYKAMRARHAYGPKGHRGMSVLIFEESAMGYLEAERLQKQFFKEGRGKDDWERRRVLFQPGGKRVLYGYLATKEEMEIFNRHSKGKARLKYDMKSYHKMVVEPMKQMDEDNQKLTWFKTKVAKQQEHSKTLEETVSVVASKLRMKDTEIKIIRQRATEQHEESKKEMDYLEQSYKQQIDQLNEDIAKREFELENMQE
