# Supplementary material for: TAS3 miR390-dependent loci in non-vascular land plants: towards a comprehensive reconstruction of the gene evolutionary history
Source: PeerJ. 2018 Apr 16;6:e4636. doi: 10.7717/peerj.4636 (PMC5907777; doi:10.7717/peerj.4636)
Supplement: Figure S6 [file peerj-06-4636-s006.doc]

**Figure S6.**

**SGS3 amino acid and nucleotide sequences in charophytes.**

Amino acid signature typical for RNA recognition XS domain is in brown. Initiation codon is in yellow; termination codon is in green. 1KP accession numbers are shown above the protein sequence.

***Interfilum paradoxum* (class** [**Klebsormidiophyceae**](https://www.ncbi.nlm.nih.gov/Taxonomy/Browser/wwwtax.cgi?mode=Undef&id=131220&lvl=3&lin=f&keep=1&srchmode=1&unlock)**)**

**FPCO_2030239**

gtcctcatgctgccaagaacttgaagtggatgcaaggtagcttagctcctaggagaatgt

V L M L P R T - S G C K V A - L L G E C

ggtaggagtttagcagaatgcagctctgatgctcgtgcgctggggcaataggcaccctgt

G R S L A E C S S D A R A L G Q - A P C

gatcgcgcggcgcttctcaccatggctggaaacggttgggacctgcctgcgggagccagg

D R A A L L T M A G N G W D L P A G A R

ggccccggttggcagtggtcagagcccgccccccaggagcgcagctggaacaacagtgcc

G P G W Q W S E P A P Q E R S W N N S A

gtgccgcagcaggggggatctgggaccgtgagccccactcttgcctgggacaatgggaac

V P Q Q G G S G T V S P T L A W D N G N

gccccccccacccgaggctgggatgcggggagctcccagggagggggaatgagacagggc

A P P T R G W D A G S S Q G G G M R Q G

agagtgagcccccagccgagcacctgggagcagtggggcgaaggggggggcagagagttt

R V S P Q P S T W E Q W G E G G G R E F

gccgccgggggggcggttggaacccctggtaacaaaccgaaaggccacggctggcaggac

A A G G A V G T P G N K P K G H G W Q D

cccaatgccagggagtttgtgcctgcgcaccccccccacgcaccatatcgctcctcgggg

P N A R E F V P A H P P H A P Y R S S G

gttggccacacccccccagacacgccaggcagaggctgggaccttcctcctgtgagcgtc

V G H T P P D T P G R G W D L P P V S V

gggggggggagtgcagcaggctggcagagccccaccctgcctcagaacaattcctggggg

G G G S A A G W Q S P T L P Q N N S W G

agccagcacagcatgagccccactgccaggcagcctgggtccccctcctgggaggacgag

S Q H S M S P T A R Q P G S P S W E D E

gcgctcaaagcgaagggcagggttgctgcatttgggaggggggttgggatggcaccactc

A L K A K G R V A A F G R G V G M A P L

agaccccccgggaacaggggcaagagggggccagtatcggctgcgccggtgtacaagggc

R P P G N R G K R G P V S A A P V Y K G

cccattcccaccaacagaggctggggcttcgagaagcaaacgatgctcaagacggacgac

P I P T N R G W G F E K Q T M L K T D D

aagcgcgcctatgaggactatgagagctacaggaccgcaggatccgagttcagcaacagg

K R A Y E D Y E S Y R T A G S E F S N R

ggagggttcaaccctgggtatggaaatgtcccccccgatgagcccatccacatgccaatg

G G F N P G Y G N V P P D E P I H M P M

catgtcagggaggctgcctatggcgcctttcctcgtccaggctcagccaactctgttgga

H V R E A A Y G A F P R P G S A N S V G

agtgggggtcgagatccaggatacgatccccagcttatgcagtttgagagggggtatggc

S G G R D P G Y D P Q L M Q F E R G Y G

ctccctggggggggtcgaggggggggttggcctggcgggcggggcgggtttgagggtggc

L P G G G R G G G W P G G R G G F E G G

aatcgaagcagggctccagcttatgtgcctgagggtttgaactgggaggggaggagctct

N R S R A P A Y V P E G L N W E G R S S

ggctcaaatctgagcaggtacgaggacgccagcagccagttctacaaccggggggttcct

G S N L S R Y E D A S S Q F Y N R G V P

ccgtgggggaatgctgcccccctgcagggtttcagggatggtgactatcccagcatggtt

P W G N A A P L Q G F R D G D Y P S M V

gggagcagcgggatggggggcagtgacagcgagagcgatgagctggacatcgcctctgac

G S S G M G G S D S E S D E L D I A S D

gatgagctggaagggtttgagggtttggccaaagagaaggaggagaaggcgtacaagaag

D E L E G F E G L A K E K E E K A Y K K

tacaagcgggtgtacctggagttgctccaagaagcaggcaaaggcgatggctccaaggac

Y K R V Y L E L L Q E A G K G D G S K D

cagtggaagtgccttgtctgcaagaaccgcaccaagttctggttcaacagtctgcacgcg

Q W K C L V C K N R T K F W F N S L H A

ctgcaccagcacgcagagaccaagaagaatgagtggcgcatccagcacaggggcctggca

L H Q H A E T K K N E W R I Q H R G L A

cgagcgctgcgggagcttctgagcagagatcccatggggccgcttggcattcacctccct

R A L R E L L S R D P M G P L G I H L P

cctgttgaggacgacgaccagatcattgtgtggcctcctgcggttattttgcaaaacacg

P V E D D D Q I I V W P P A V I L Q N T

cgcgtcaggatggatgctgagggccactggaacggcctgggcgccactgagatccgcgag

R V R M D A E G H W N G L G A T E I R E

cagtacaagagcttcaaccccatggctgtgcgccctgcttacggcccagacggccacaag

Q Y K S F N P M A V R P A Y G P D G H K

ggttgctgcgtgatccaattctcagcggaccccaatggctttgacaaggctgtccagctg

G C C V I Q F S A D P N G F D K A V Q L

agccagtactttgagaagcagagcaggggcagagaggcctgggacgtgcatggaagccgc

S Q Y F E K Q S R G R E A W D V H G S R

agcaagcgcttcgatgacgatggcaagctgctcgtgtttggctacctggctcagccaaag

S K R F D D D G K L L V F G Y L A Q P K

gatatgtggcagctggacaagtttgccaagggcaagaatggcaaggcggggcagctggtc

D M W Q L D K F A K G K N G K A G Q L V

aagtggtggactgagagcaagaaggaagcggtggacgatgagaaggtgcgccgcgagcag

K W W T E S K K E A V D D E K V R R E Q

cagcgcctgcaggaggcgggtgaggtcagcaggctgcgtgagaaggcagctgaggatggc

Q R L Q E A G E V S R L R E K A A E D G

gacgcgcgcgcgcgcgcggagagcgagctgaaggtgcagtccaagaaactgcgcaacctg

D A R A R A E S E L K V Q S K K L R N L

caggagcgctacaccaccgtgcagcagctgcagcagcagcagctgggccagcacaaggag

Q E R Y T T V Q Q L Q Q Q Q L G Q H K E

gagctggagtccctggcgctgttctacgatggcaagttcaaggagatgggcgagcagttc

E L E S L A L F Y D G K F K E M G E Q F

acagtcaccgagcaggacgcgctggccaagcgcaaggaacgagagacagccgatgccaag

T V T E Q D A L A K R K E R E T A D A K

aagctcagcgccctggagaccctcaaagacggcactgaagaggaaggggaaaaggccaga

K L S A L E T L K D G T E E E G E K A R

ctggagcagcagatggcctctttggaagaggagattgccaccaacctcacgctggccatc

L E Q Q M A S L E E E I A T N L T L A I

aagcacaaggacgctctcgggcaaatggaggccacacacaccgaagaacttctagagctg

K H K D A L G Q M E A T H T E E L L E L

cgcaagcgtcatggagaagaggagctcactcttcgcaagaagcagcaggagcaaaaggcg

R K R H G E E E L T L R K K Q Q E Q K A

gcgctgctgcagaagctcgaggtgagcgcgcccgcgcgcgctctctctccggacgcctcg

A L L Q K L E V S A P A R A L S P D A S

gacaaaggaaagctgtccaccgaggcgcccgagttcaagccaaagggcgcagaggaagtt

D K G K L S T E A P E F K P K G A E E V

gcgcaggactcggacacagagaagaaggctccggacgtcaaagcagatgacacccatgcg

A Q D S D T E K K A P D V K A D D T H A

gagctggtcgggaagatggttgacgacgtgctttcggacgaggggggttcggagaaggct

E L V G K M V D D V L S D E G G S E K A

gcgagcgagaagccggggagccccccccccgaggagaagccgtccgaactcgcaaaagtg

A S E K P G S P P P E E K P S E L A K V

tccgaagcggaggtcatgagcccgctgccgaagctggcgaaaggcccactcaccaagtcg

S E A E V M S P L P K L A K G P L T K S

cgctttgtggtgctggaagacacccctgagccacagaagactgccagcaaggcgtccgac

R F V V L E D T P E P Q K T A S K A S D

atgctgcccaggacgctctccctcgaggacccgtccgacttgacagctggcgcgagtccg

M L P R T L S L E D P S D L T A G A S P

cagggggggttccgtgtgtacaaaacccccgagcaagacggctcaggcaatctgcagcag

Q G G F R V Y K T P E Q D G S G N L Q Q

gcgattgagcgcctgagcgctaatggggggggggagaagagtcccatgttcaacaacagg

A I E R L S A N G G G E K S P M F N N R

gccaatagcgctgggggcgcattcaatggcgtcctggacgctttttcgggcgggtgggag

A N S A G G A F N G V L D A F S G G W E

gcgcctgctgcgtccgcattagacggggggtccgcttcttgggctgacgtggccaagaag

A P A A S A L D G G S A S W A D V A K K

gaggcggaaaagaaggcaaaggagagtggagctctgcctgctggacagctggcagggagc

E A E K K A K E S G A L P A G Q L A G S

tagttggtgatctggtttggttaattggaaaagagcattttgtcagaggcctgcccgttt

- L V I W F G - L E K S I L S E A C P F

ggaggtttggtgtagtgagagtcgagaaggagagggcatttcgagcaggtctgtcaggga

G G L V - - E S R R R G H F E Q V C Q G

atggatgcccatgcccctgcgcagatcctgcagcacacgtcacgagatcgattctctgga

M D A H A P A Q I L Q H T S R D R F S G

gaggctgctcttggagaggcttaaattcaagtcaaatagcaggcgcacactctgcggggg

E A A L G E A - I Q V K - Q A H T L R G

***Entransia fimbriata*** (**class** [**Zygnemophyceae**](https://www.ncbi.nlm.nih.gov/Taxonomy/Browser/wwwtax.cgi?mode=Undef&id=131209&lvl=3&lin=f&keep=1&srchmode=1&unlock))

**BFIK_2027377**

gtttggaccaggcaccccgtgaactgcaggtgctcacctgtgcataccccccactgagcag

F G P G T P - T A G A H L C I P P T E Q

agataaggatcaatgcgagctgcacgctgctcatactgagctatcttgaagccctggttg

R - G S M R A A R C S Y - A I L K P W L

caaatgagctggtctatggtgacaagccagcgagggaggggcaatcgtggctggaaccag

Q M S W S M V T S Q R G R G N R G W N Q

ccctttgacagctcatcttttggtcccgatgtggccagcaggacctccgcatccagcttt

P F D S S S F G P D V A S R T S A S S F

gaggattggggcagcagtggccctccaagtcatggtcttggcagccagtcatgggcggat

E D W G S S G P P S H G L G S Q S W A D

gaagtagagtatgcagaagtagaggatgcagaaagtgacgatgactactccgacgtctcg

E V E Y A E V E D A E S D D D Y S D V S

gacgaggactcgaacgaagttgtcagtgaaatggtgtacgagaagttgaaggagcggtac

D E D S N E V V S E M V Y E K L K E R Y

agacgagagattcagcagtctgtcggggatggtccccgtcagtggccatgccacgtatgc

R R E I Q Q S V G D G P R Q W P C H V C

aataccgagaacaagacgaggaccaagtttgccaaaggagaggagctgcagcagcatgcc

N T E N K T R T K F A K G E E L Q Q H A

tccaagtgcaagaagagggtggcagcccacaaggggcttggaagggcattggccgaggct

S K C K K R V A A H K G L G R A L A E A

atgggctcggagcagcctgtggtgccttcggagccacgtgcggtggtgcccaccagcaat

M G S E Q P V V P S E P R A V V P T S N

aagacgattgtctggcctccgatggtcatcgtccgcaacacacggatagagcaggagaac

K T I V W P P M V I V R N T R I E Q E N

aacggcaagtggggtggtttggggagcccagagctccgcctccaactggccgacttccac

N G K W G G L G S P E L R L Q L A D F H

actgagaaggtgaagcatcattatggaccccgaggccacatgggcgtcacgttggtggtt

T E K V K H H Y G P R G H M G V T L V V

ttcgaggccacgccgaccggctattacgaggccaagaggctggcagaggagtttgctaaa

F E A T P T G Y Y E A K R L A E E F A K

gccgacctggacagagaggggtgggctcaggtggggagtcgaacgattcggaagacccgg

A D L D R E G W A Q V G S R T I R K T R

gacgggctgaacttgctgtatggctacctggccgaagagcaagacatggcccaattctcg

D G L N L L Y G Y L A E E Q D M A Q F S

gaccaccagaagaagaagaagcctctcgtgccatggaggatggagaggttgcaagagaag

D H Q K K K K P L V P W R M E R L Q E K

gtgatcgatccaccgcgccgggtagccatcgaggcggtggctgtgcaggaggacaacgcc

V I D P P R R V A I E A V A V Q E D N A

cggctcctgaaggacaaggaggagcttgccaagaagctggaggtggtcaccgacgacaaa

R L L K D K E E L A K K L E V V T D D K

ctgagggtccaagagctggtgcagggcgtcgaacaggacctgaggctggcccgtggtgct

L R V Q E L V Q G V E Q D L R L A R G A

cgagatcaacggcaggatcatatcgattccctggtaaagaagatccgggacatggaggag

R D Q R Q D H I D S L V K K I R D M E E

atatggcagcgggatacgcaggcgctgcaggatgagatagaggtggtgcggcaggagcgg

I W Q R D T Q A L Q D E I E V V R Q E R

gcccagtttgcagacgaccgtgaccgccaccgggcggaggccgagcggctgcagcagctg

A Q F A D D R D R H R A E A E R L Q Q L

ctgagggtcagggatgcggaggtggcagatctacaggagctgctggggcgggcg

L R V R D A E V A D L Q E L L G R A

***Planotaenium ohtanii*** (**class** [**Zygnemophyceae**](https://www.ncbi.nlm.nih.gov/Taxonomy/Browser/wwwtax.cgi?mode=Undef&id=131209&lvl=3&lin=f&keep=1&srchmode=1&unlock))

**SNOX_2003373**

aggggggaggagaagcgggggcgctgcccaaacgcggtttggttgacaagagcgatggtgaa

G G G E A G A L P K R G L V D K S D G E

gccgaacatgatgacgtggcatttttcgtcggcgatggaagttcagagaatgcttcgtac

A E H D D V A F F V G D G S S E N A S Y

gacgacgaggacgggttgtacgacgaggacgacgaattcgaccctgataacgtcaatatc

D D E D G L Y D E D D E F D P D N V N I

gagacagagagcgattcgtccttcgtggagaaggagagaggagaaagggaggcagggggg

E T E S D S S F V E K E R G E R E A G G

gagttcgtagggttgagcttgaaagagatgcaacgagagacgcttcccgcccagtggagc

E F V G L S L K E M Q R E T L P A Q W S

gatgaccccgtgtcgcgttacgtccgggaatggcaggaagacgcggcgcgctgcgncagc

D D P V S R Y V R E W Q E D A A R C X S

ctcactggcacggcggacgatttgtcttccctgccctcgtctgccgtgaagacggacatc

L T G T A D D L S S L P S S A V K T D I

atggtagtcagtgagagtggacacgactcggaggcgggaacagagggagtaagggaggcg

M V V S E S G H D S E A G T E G V R E A

agagtcttcgtcctcggagcgcgcctcccttcccttcaggacgcgggcgatctgctggag

R V F V L G A R L P S L Q D A G D L L E

ggagagggagagaaagagggtgcgaacgaaggagaggatgccttgagcgtctctgcactc

G E G E K E G A N E G E D A L S V S A L

tccgatgcgtattcagaatttgacccgagagagaaccccgtggagtttccccactctcag

S D A Y S E F D P R E N P V E F P H S Q

cccgtcttccacttcttcaaggaggtgatggggatggaggaggaggagctgagagacaac

P V F H F F K E V M G M E E E E L R D N

gagagagagtacaagtgccgggtgtgcgtcggggggaaaggggagattgcctggtatcaa

E R E Y K C R V C V G G K G E I A W Y Q

ggcgtccggtcgctcatccagcacgcgtccaccatcaagaaccgaaaggtgaaagagcac

G V R S L I Q H A S T I K N R K V K E H

cgcatgttcgcccgctgtctggagctgattttgtcccaaaagggtttccaagaggggatg

R M F A R C L E L I L S Q K G F Q E G M

tggggagagattcccaagctgaggatgtggaaaggtctgggcaaggctgccgcgagcaaa

W G E I P K L R M W K G L G K A A A S K

gggaaagaggaggagttcatcgtgtggccgcccgttgtgatcatcagaaacacgcagctg

G K E E E F I V W P P V V I I R N T Q L

ggactcatggaggacggaaggtacactggaatgggcagcaaagagctctctgattatttt

G L M E D G R Y T G M G S K E L S D Y F

ccctgcaagcatatcatgaaggcaaaacacgcctacggaccgcagggccaccgcggcatc

P C K H I M K A K H A Y G P Q G H R G I

tccatgcttgttttctctccgtcccttcctgggtaccaagaggccctcgacctgagcaat

S M L V F S P S L P G Y Q E A L D L S N

actttctcgcaccaaaggagagggagagaggagtgggagagacaaaacgggaagagagtc

T F S H Q R R G R E E W E R Q N G K R V

gacaaaaacgggaagctggtattgtttggatacatggcgaacgcggcggacatggaggag

D K N G K L V L F G Y M A N A A D M E E

ttcgtgaggcatagcgggaagaacgagagaaaggtcgtttcctacgaaatgaaaaagcgc

F V R H S G K N E R K V V S Y E M K K R

agagaagtcgttgatcgccctttcaaaattttgatcaagaaagcggatgaatcggccaag

R E V V D R P F K I L I K K A D E S A K

gttcaggagaagctctcggacgtgtcccagcgctggaaaaatcagctagttcattcttct

V Q E K L S D V S Q R W K N Q L V H S S

cagctcgaagccgtcgtggagcaagagaggagagagcgagagagggagagaggggagaga

Q L E A V V E Q E R R E R E R E R G E R

gagaggctaatggccagactgaaggagacgaaggaggagtcgcagaggaaggagagggag

E R L M A R L K E T K E E S Q R K E R E

aatgaagcgctgaagcaaaaatatatggagctacttagcaagcatgaggaagagctaagc

N E A L K Q K Y M E L L S K H E E E L S

gagatggacagaaggcaccactccctcctaatggaaatgagagcgctggagagaaagaga

E M D R R H H S L L M E M R A L E R K R

gaagacgaggagatgaagaggatcgagagggcgaccgaggagcaaagaaggaggagggag

E D E E M K R I E R A T E E Q R R R R E

gaaatcagggagagcagctgcacggactccgaagtggacgcagcgcgtagcgaggccctc

E I R E S S C T D S E V D A A R S E A L

ctccagcgctcggagcgcgatctatccgactactcggagcgcgtcgcgcggctcgaagaa

L Q R S E R D L S D Y S E R V A R L E E

gcctttcacgcgcaactgaaaggcgcccgcgaggagcgtattgcgttcgagctatccctc

A F H A Q L K G A R E E R I A F E L S L

cgcagcgctcacgaaggagagagaaaacggctggcagaggagtatgaggggatgtttatt

R S A H E G E R K R L A E E Y E G M F I

cagcaaggggaagtctttggtaagatgatgagagagaaggagagagggagagggggggaa

Q Q G E V F G K M M R E K E R G R G G E

ggggggagaggaggaaagggagcgggagacatggttggtggagctgaaaaaaaaagcggt

G G R G G K G A G D M V G G A E K K S G

ggaaccgagagggggggagggagagacgatgagggagggcgaggggggcaaggtgagagt

G T E R G G G R D D E G G R G G Q G E S

gaaagacacgaggaggacctgggagatgagaagaacaggggagaggaaggaggggagagg

E R H E E D L G D E K N R G E E G G E R

***Staurodesmus omearii*** (**class** [**Zygnemophyceae**](https://www.ncbi.nlm.nih.gov/Taxonomy/Browser/wwwtax.cgi?mode=Undef&id=131209&lvl=3&lin=f&keep=1&srchmode=1&unlock))

**RPRU_2010384**

ggattgggaggctgagagtgtggtatcctctgtgttcccttctgtggatgtgactttgcct

D W E A E S V V S S V F P S V D V T L P

ggaagaagggatgaggggcatgggagctggaagcccagtggttctgtggaccagtcttcc

G R R D E G H G S W K P S G S V D Q S S

ctgcaggaggccaacttggaggctggtatccctggtgtacctgctgtacctgctgtacct

L Q E A N L E A G I P G V P A V P A V P

gtgggctttcctgcatcggtcaaatccggtgtttctgtcaaatctggtgcatctgggagg

V G F P A S V K S G V S V K S G A S G R

tctggtgttcctgagggtgttcaatcgagcacttcaggggtggctgagggtacacctggg

S G V P E G V Q S S T S G V A E G T P G

ccaaacataggccctcattctccccatggaattcatggtcctcaggaggttgtgcctggg

P N I G P H S P H G I H G P Q E V V P G

gacgactctttgtttgaagacaagaagggccgtacatatgattggttgcccgccatatca

D D S L F E D K K G R T Y D W L P A I S

gcagacgcagaggcagcagaaggtgatggtgcgtcagagagaggtggtcgtgctagtgct

A D A E A A E G D G A S E R G G R A S A

cgtccggcttctggtcgtctccatgcactctctgggttagaggcaactgttggctctgaa

R P A S G R L H A L S G L E A T V G S E

gggctgacagtgggaggtggagcaggtggtggggagagagtggggggaggtgtacctcta

G L T V G G G A G G G E R V G G G V P L

tcttaccaaggggggagtgagagagtgatggcgggtgcacccttgtcgtaccaagggggc

S Y Q G G S E R V M A G A P L S Y Q G G

attgaggcgtttgaggatgatgaggatgatgaggaggaggaggggatggagaccgacgac

I E A F E D D E D D E E E E G M E T D D

gacaacgaaagcgatgctggcgggaaggaaggcgcgaggaaggacctcttcatggtggac

D N E S D A G G K E G A R K D L F M V D

aacctcctgaaggagctgcaggagctgccagttccggagatccttgagaggatctggaag

N L L K E L Q E L P V P E I L E R I W K

tgcccggtgtgccagcctgctgccataggggaggtttgtaaggggcacaccacttggtgg

C P V C Q P A A I G E V C K G H T T W W

ccgggcatccgatccctccttcagcacgccaggaccaagtccatcaagaaacgcaagcat

P G I R S L L Q H A R T K S I K K R K H

gtgctgtttgccgatcaggtggccagcattctggcgcaccgagagatcatagtggcgggg

V L F A D Q V A S I L A H R E I I V A G

ggtcctacgcctggggatgggagtgttagtaagtactggaagggcgtaccggggaaactg

G P T P G D G S V S K Y W K G V P G K L

gagaaagcaccaaactccattgtcatgtggccccccactgtcattgtccggaacactcag

E K A P N S I V M W P P T V I V R N T Q

cttgaccagagagacgatgacggaagggtggtggggatgggcagtcaggagctgcggaat

L D Q R D D D G R V V G M G S Q E L R N

cggtacaaggaatgcaaacatctctccaaagccaagcactcctacggtccacatggtcac

R Y K E C K H L S K A K H S Y G P H G H

agaggcatgagtgcgttgcaattcgcgtgcacgccccaagggctgagggaggcggagaga

R G M S A L Q F A C T P Q G L R E A E R

ttggctgatcagtttgataaagccgataggaacaagtactcctgggtgaacaagaccaaa

L A D Q F D K A D R N K Y S W V N K T K

cccaaagtggatagggaagggaacaggctcctctttggttacctggcagaggcttcggac

P K V D R E G N R L L F G Y L A E A S D

attgcagacttcaataagtgccagaaagggaagcaagccctgaaggcagaaccgaaaagg

I A D F N K C Q K G K Q A L K A E P K R

aggggggaagtgatggcaatggaggagcaagagaagatgaatgccaagatttggcaggct

R G E V M A M E E Q E K M N A K I W Q A

caggctatcgatgcgtctcataagctgaccacagagcgggtgaagtcagcgcaaatgaag

Q A I D A S H K L T T E R V K S A Q M K

tcaaagtttgaggaggtggaagaggagaagcagcagctggcccagcagcttgagcatctg

S K F E E V E E E K Q Q L A Q Q L E H L

gagaggaaattcaaagtgaaaggggaggaagtgaaaaaactccgagagagaaatgcgcaa

E R K F K V K G E E V K K L R E R N A Q

atgtcccgcaggcaccaggaagagatggaggatttggaggacgcgtacgataagcagctg

M S R R H Q E E M E D L E D A Y D K Q L

cagcgcatagccctgcagaggcaggcgactcagagcagggaagcacagctgcaggagcgg

Q R I A L Q R Q A T Q S R E A Q L Q E R

actcagaaggcaagggcacagcacatctctgctatgagtgaagcagagagagagacccat

T Q K A R A Q H I S A M S E A E R E T H

tcccatgctggctctggccattcacaccggggatctgcctcggagaaggagtctgggagc

S H A G S G H S H R G S A S E K E S G S

ccatctaagatgcaattgaggagtcggatggaagctgcagtgcaaggcagcttcaacgac

P S K M Q L R S R M E A A V Q G S F N D

gaggcggcgtttgcgaaggagcagcatgatctggaggaggagcgccacaagcagcttgct

E A A F A K E Q H D L E E E R H K Q L A

catctcagaaggcgcttccaggaggaggagctgagactggagacggagttcgagaagagg

H L R R R F Q E E E L R L E T E F E K R

agagcggccattctggtgctgtatgaagagcggcagaggaaagcggagagcgatgtgctt

R A A I L V L Y E E R Q R K A E S D V L

gggtgggaggatgaggaggatggcagcacaggagggactgagtctgctgtgggtacaccg

G W E D E E D G S T G G T E S A V G T P

gtggcagagcatgtcgtggggggaacagcaggcagcactgcagagggggagaatgcagga

V A E H V V G G T A G S T A E G E N A G

agagcaggaggagctgagggcactgggggtacaccggtggcacagcatgacctgggggga

R A G G A E G T G G T P V A Q H D L G G

accgcaggcaacactgcagaggatgagaaggatggtggcggaggaggatctgagcgtgct

T A G N T A E D E K D G G G G G S E R A

gcgggtacattggtgagagcagagcatgttgtggggggaactgaagctggcgctgcaggg

A G T L V R A E H V V G G T E A G A A G

ggagagaatgcaggcagagcagggggagctgagtgcactgggggtacaccggtaagggca

G E N A G R A G G A E C T G G T P V R A

gagcatgctgggtggggaactgaagctggcatcacagagggggagaatggagtcagtgga

E H A G W G T E A G I T E G E N G V S G

caggtggtgggggttgggacagcagaaggaatggacactcctgtgggtacaccgctgacc

Q V V G V G T A E G M D T P V G T P L T

ccacggggctctgggggtacaccactaaccccntcagggctctgggggtacaccactaac

P R G S G G T P L T X S G L W G Y T T N

cccacggggctctgggggtgcaccacagccagagtatgctgcagagagagtagatgctag

P T G L W G C T T A R V C C R E S R C -

tagtgcagtggaggagtgtgaaaacagtggcgaggtggtgggaggtgcaagaggggaaat

- C S G G V - K Q W R G G G R C K R G N

ggaggatgtcgagggtgcgccagtggcaggggacgttaggggaacagccgaagtgagaag

G G C R G C A S G R G R - G N S R S E K

tgtagtggattgtgaagggagaggtgagagagaggtggcagaggatagaagggtggagga

C S G L - R E R - E R G G R G - K G G G

agataggagggtagaggaagataggaagttggaggaag

R - E G R G R - E V G G

***Cosmarium ochthodes*** (**class** [**Zygnemophyceae**](https://www.ncbi.nlm.nih.gov/Taxonomy/Browser/wwwtax.cgi?mode=Undef&id=131209&lvl=3&lin=f&keep=1&srchmode=1&unlock))

**HJVM_2002356**

cagatgcggagactgttgggaccaggtccgaggtctcaacactcccagcttctgactgcctg

D A E T V G T R S E V S T L P A S D C L

ccctccatctctggattcgaattctcagactccttctctgacattgggtcagagtcgcct

P S I S G F E F S D S F S D I G S E S P

tcaactcttggcacgccactgccctcagcagccactgagagcatgccaannnnnnnnnnn

S T L G T P L P S A A T E S M P X X X X

nnnnnnnnggttggagctgttggagtgggagggaatggagaggagggggaaagggacggg

X X X V G A V G V G G N G E E G E R D G

gggatagaggaggaggacgaggacgaggaggacgagagtgaggatgaggaggtttggggt

G I E E E D E D E E D E S E D E E V W G

gattggatgaagaagacactgaatgaccttgaggagcagctgcagaagatgagcgcaatg

D W M K K T L N D L E E Q L Q K M S A M

caggtgctcaacatggagtggaagtgtccctgctgtcagcccaaaaaaggggagacggtc

Q V L N M E W K C P C C Q P K K G E T V

tgcaagggctcaacgacatggcacaagaacctgaaagccatgctccagcatgcgcagaca

C K G S T T W H K N L K A M L Q H A Q T

aagcagaagcgtatccgtctccacagggacttcgcctctaggctcgaggcgctgctgctg

K Q K R I R L H R D F A S R L E A L L L

cgcaaggggatcaaccttgatcctggcctgcctgggcacgtggccggcacccggctggtg

R K G I N L D P G L P G H V A G T R L V

tctgggcactgggtggaggcgcctgcctcaagagagcacgacccctacgtgatgtggcct

S G H W V E A P A S R E H D P Y V M W P

cccaccgtcatcttccgcaacacccagtttgatcttagagatgatgatggcaagattact

P T V I F R N T Q F D L R D D D G K I T

gggatgagcacgcaagagctgaaggaaaagcataaagagtacaggaacatcactaaagtt

G M S T Q E L K E K H K E Y R N I T K V

agagcagcttatgggcaccagggccaccgggggtggagcgctgtccagttcgaagccacg

R A A Y G H Q G H R G W S A V Q F E A T

atgcaaggcctgcaagaggctgagagcctcgcggctacgtatcggagagtgaacagggac

M Q G L Q E A E S L A A T Y R R V N R D

aaggctggctacaacagcagccacgttcccaaggttgaccgcaacggaatgcgggtgctc

K A G Y N S S H V P K V D R N G M R V L

ttcgcatacatggcagaagggacagacattgccgagttcaaccgcagcgcgaagggcgcg

F A Y M A E G T D I A E F N R S A K G A

caggcgctgaaggcaactccgaggaagcgcagggagatccaggcagcgtaccagacgcag

Q A L K A T P R K R R E I Q A A Y Q T Q

ctggatgagaagatgcagcaggccaagctcatggagcagcaggccatccaggtttcacac

L D E K M Q Q A K L M E Q Q A I Q V S H

cagctggtggaggagaagacgcgctctgcgcagctcaagttatgcctgaaagaatcggag

Q L V E E K T R S A Q L K L C L K E S E

gagtcgagggctctgctggaaaaggggtacgagaagcttgagagggagctggccaataag

E S R A L L E K G Y E K L E R E L A N K

agggctgagatgcagcgaacaaaggagaagtctgctgccaatgctgcgagggtgaagggc

R A E M Q R T K E K S A A N A A R V K G

gaggtggaggactcggagaggcagttcttgaagcagcagcaggagatgcaggaggagagg

E V E D S E R Q F L K Q Q Q E M Q E E R

gagacgttccggaggagagaagctgagttgcagaggaagcttgaggagatgaggagggac

E T F R R R E A E L Q R K L E E M R R D

atggtgcaccgaatggtccagcgcgaggggcccgtggcagcagcagaaaagcctacagag

M V H R M V Q R E G P V A A A E K P T E

gaggctgcctttctcgcaggcgagactgcgtctgctatcgatgtccatctgagcgagttg

E A A F L A G E T A S A I D V H L S E L

agggagacggcgctccaggacagtctgaaggacgaagccgagtacgtgggtgctcaggag

R E T A L Q D S L K D E A E Y V G A Q E

gacttgggcgcagaggagcagaggacgctgcagttgctgctgaacaactatcatgccgat

D L G A E E Q R T L Q L L L N N Y H A D

gtgctcgacgtgaagcgacagtttgcagcgcgtagagtggccctcctggaggcgtacaag

V L D V K R Q F A A R R V A L L E A Y K

gagaggcaggagaaggagatgaaagtggttgaagacgaggaggttgtgcggagcgaggca

E R Q E K E M K V V E D E E V V R S E A

gagggggaccaaaatatggggaaagaggtgga

E G D Q N M G K E V

***Coleochaete scutata*** (**class** [**Coleochaetophyceae**](https://www.ncbi.nlm.nih.gov/Taxonomy/Browser/wwwtax.cgi?mode=Undef&id=304573&lvl=3&lin=f&keep=1&srchmode=1&unlock))

**VQBJ_2006760**

gctcgcttatctggagattaccaaaagaaagaaatggtgtccgcgaatgcagctacagga

A R L S G D Y Q K K E M V S A N A A T G

ggtccagattcaagtgatgtagagtctgaagatgatgagaaaaccatgaattacgcctac

G P D S S D V E S E D D E K T M N Y A Y

aagaaatattcacaagagctttccaaacagttgactaaaaatggagctgctgaagttgac

K K Y S Q E L S K Q L T K N G A A E V D

gctcagcagccacagtgccgggtttgtaagaagacgtcaaacaggagctatcgaaattgg

A Q Q P Q C R V C K K T S N R S Y R N W

aattcactgtaccagcacgctgctaatacaacggcaattcgccccattcagcatcgagga

N S L Y Q H A A N T T A I R P I Q H R G

tatgcacgagctttggcagagtatgcccctgttgtcaagattgcagctggcccatcagtt

Y A R A L A E Y A P V V K I A A G P S V

cagcacaatggactgaagcgggacattgacgataagataatctggccgccggtcatagtg

Q H N G L K R D I D D K I I W P P V I V

ttggagaacgcctggtcagttgacccgaagatccaagcaaagaatgtgggtttgactact

L E N A W S V D P K I Q A K N V G L T T

gcgcagctcagggacttgttcaagacgagctcgccaagtgccaaggtaaaggcactgtac

A Q L R D L F K T S S P S A K V K A L Y

aggacggacaagcacgtgggagtggggctcctggtgttccccggcagccgagatgggtat

R T D K H V G V G L L V F P G S R D G Y

gtggaggcgacactcatggccgacaaattcctcaggaatggtcggggtcgcgaagcctgg

V E A T L M A D K F L R N G R G R E A W

aatcgtgaaaggcagatcagggatggtgtgtacaagggtctcaaaggcgcgagcgggacg

N R E R Q I R D G V Y K G L K G A S G T

gtcattctgtacggctacctcgcgaatttggacgacatggagaggctggacaagtcgaag

V I L Y G Y L A N L D D M E R L D K S K

cacaatgtcaagtggagaatcatgacgtacagcgaggctgtggaggctccggcgaagaag

H N V K W R I M T Y S E A V E A P A K K

aaagcccaggtggttgtacagcaggagaaggttgagaaagcagatgagcccccccccatg

K A Q V V V Q Q E K V E K A D E P P P M

gcagaagtcgaggcagtggtggacgaagcagcgagggcagaacaagaggcgaaggaggcg

A E V E A V V D E A A R A E Q E A K E A

gagagaagagcgaaggaggaggtcttgcggcggaggatggaggaacttgcgaagttggaa

E R R A K E E V L R R R M E E L A K L E

aaggagatcaaccgcctggatggtgagatggaggcactggatcagaggttctttgagaat

K E I N R L D G E M E A L D Q R F F E N

atgagaacacgggatgcacagcatgaggaggaggttgcggaattcgagagggaggaagca

M R T R D A Q H E E E V A E F E R E E A

gcgtttgacgatgagaggggagattcgagcgtagaagaaggtattacggacaagagggaa

A F D D E R G D S S V E E G I T D K R E

aaggcgctcgcaaaggaggcaaaattacgggaacagatcaagtctgtctcagactacgtc

K A L A K E A K L R E Q I K S V S D Y V

gagcccgaatctgtacccatgatggaggccatgctccagcacgtgacaggtgtcgccacg

E P E S V P M M E A M L Q H V T G V A T

ctggtatcccgggaccacgatgagatcctgaaggtgaaggctgagctgtacaccatggag

L V S R D H D E I L K V K A E L Y T M E

ctcgatctcaagcgcaagcaccttgaagagagacgcaagctcgctggtgtggcagcggcg

L D L K R K H L E E R R K L A G V A A A

aagagaaacgaggtgctgctgcgatcggccgaggcaagggaggctgcggtggacaagctg

K R N E V L L R S A E A R E A A V D K L

tttgctgaaattgagcgtcttgagacatcggggaaagccaatgggaaggacgaggcagct

F A E I E R L E T S G K A N G K D E A A

ccgattcccactgctccggcaacagtcgacgttgagatggtggaagctgttgccgatgct

P I P T A P A T V D V E M V E A V A D A

gagcttgtacaaccctaagacagaaccttgtctgtggacaagcagccatggcctgtaatt

E L V Q P - D R T L S V D K Q P W P V I

catggtggctctgcattttctcatgcagaggggagagagagagagagagagggtggtggc

H G G S A F S H A E G R E R E R E G G G

ctggctgctgaatcatcactcatcgctctgggctaccatgtacccatacccatgcgaagc

L A A E S S L I A L G Y H V P I P M R S

tctcaacaaagctggccatgaaagggaacccttatggaatccatgaatttctccaggact

S Q Q S W P - K G T L M E S M N F S R T

aattttcacagacttgtttttttgaaggcagac

N F H R L V F L K A D

***Chaetosphaeridium globosum*** (**class** [**Coleochaetophyceae**](https://www.ncbi.nlm.nih.gov/Taxonomy/Browser/wwwtax.cgi?mode=Undef&id=304573&lvl=3&lin=f&keep=1&srchmode=1&unlock))

**DRGY_2003110**

tgatgcatggatctccgacgcactggacttcccactaaaataagctgcatggatcagacg

- C M D L R R T G L P T K I S C M D Q T

gaggaaggaaaggctttagacaagtgcgcatgcactgagctttccctaactttccctctc

E E G K A L D K C A C T E L S L T F P L

acaagttcttctttcattttttgtttttgctcaagagcacaatcaatggcgactcctcag

T S S S F I F C F C S R A Q S M A T P Q

gatgaattgattgtgtggcccccaattgtaatcatatgcaacacagcaactggatgggat

D E L I V W P P I V I I C N T A T G W D

gaggagaaggggaagataactggtatcggcaatcccgaagtggaagaccttttcaaggag

E E K G K I T G I G N P E V E D L F K E

tacttgccagtgaatggcaaaagtgcttatgataaatatggccatgcgggcataagtttt

Y L P V N G K S A Y D K Y G H A G I S F

ctcacattccacgatgatgatcgtgggtattacctggccacgttcctagacaaggcattg

L T F H D D D R G Y Y L A T F L D K A L

caagatgagaatcgggggagaaaggagtggttacgcactcgcaagagccagaactactac

Q D E N R G R K E W L R T R K S Q N Y Y

cgggtcaacgaagaggggaagcggatcctctacggctacctggcgacggtgaaggacatg

R V N E E G K R I L Y G Y L A T V K D M

ctcgacccggagatgatgaggttggcacacaaaacagtcaagtggaagaaggtgtccaag

L D P E M M R L A H K T V K W K K V S K

caggccaaggtgcacgagccggagcagtggaagctcgagctgaacaacgagatcttgaag

Q A K V H E P E Q W K L E L N N E I L K

ctgcggaaggagaaagaggagcacgaccaagaggtggccatgaagaagttggagatggac

L R K E K E E H D Q E V A M K K L E M D

aagaagttggcagaggagatggagaagcacgagaaggatgtcaagagaaaggagcaagaa

K K L A E E M E K H E K D V K R K E Q E

cacaaggacttggttgagtctaccactaaagagttcaaaaagagaatcttagcatttgag

H K D L V E S T T K E F K K R I L A F E

gagaagcagaacttggacaagcgcaaggagaaggaggaacgtgcccggctgcaagggcta

E K Q N L D K R K E K E E R A R L Q G L

ttggagcggcaggagagggatctgcttcagcagcggcttgaactggctgggcggcaaatg

L E R Q E R D L L Q Q R L E L A G R Q M

caattcgagagccgttccgacaaactgaaggagaagcgactccagctggacgcagaggag

Q F E S R S D K L K E K R L Q L D A E E

aaaaagcgggagcagcactacgcggatcgggaactgcagagggagaaggaaaagcaagag

K K R E Q H Y A D R E L Q R E K E K Q E

aagttcaagaaccgtgtgaaggagctgctgcagaagagagagagggacaagtacgaagag

K F K N R V K E L L Q K R E R D K Y E E

atgaagcagcggacggaggagcgggaggcggcgctgcggagtctggacgacgagatccag

M K Q R T E E R E A A L R S L D D E I Q

tcgcaggagagccacgtggagcagctgaagcaagacgtggcggccgaggaagcgaagagg

S Q E S H V E Q L K Q D V A A E E A K R

gaggagctgcgggagcaggagaaggaggcagagaggcggctggcgtcacagcggcaggag

E E L R E Q E K E A E R R L A S Q R Q E

gaacgctcgaactgcgccctttgcactacccccttcgcagagcgcgactggcggcctgcc

E R S N C A L C T T P F A E R D W R P A

tacacctgtcagtcccacgggcccgtggtctgcatggcgtgtgccctggaggccttcaag

Y T C Q S H G P V V C M A C A L E A F K

aagagtgagcagaaggnnnnnnnnnnnnnnnnnnnnngccccggctgcaagtcagaggag

K S E Q K X X X X X X X X P G C K S E E

tggttgcatcagctgccgtcactgaaggccaagagagcccggccggcaaagaagtcggtc

W L H Q L P S L K A K R A R P A K K S V

aaccctacggtgaaggcatgcaccaactgcaagcggtgcgacaaaaaatttgcaagcaag

N P T V K A C T N C K R C D K K F A S K

gactatcggagaggtctgccaccatgcgggcatagcattgcctgcatggcttgccttgca

D Y R R G L P P C G H S I A C M A C L A

ggagaggacaaatgtcgcctttgtaatcatcgcatcagcgcgatccttccatgccccgcc

G E D K C R L C N H R I S A I L P C P A

aaggtttactttggtgctggatgatgtttgtacatcgccacgtactacacgctatggcct

K V Y F G A G - C L Y I A T Y Y T L W P

gatctatgtgtatattgtacgtttaggtttctgtggagtgcaacctccccgtggttgaac

D L C V Y C T F R F L W S A T S P W L N

aatgtaccttgagacttgcatgcgacgcttgtaatattcttgtagtttgtcacaatgcga

N V P - D L H A T L V I F L - F V T M R

cacttgtagtaatcttgtaatctatcacaatttgttgggtccga

H L - - S C N L S Q F V G S

***Coleochaete irregularis*** (**class** [**Coleochaetophyceae**](https://www.ncbi.nlm.nih.gov/Taxonomy/Browser/wwwtax.cgi?mode=Undef&id=304573&lvl=3&lin=f&keep=1&srchmode=1&unlock))

**QPDY_2030183**

gtgccttggcagttatcttggctgtccaatcatcgcacttatcattacaagcagacctgcat

A L A V I L A V Q S S H L S L Q A D L H

ctttactagtacagcgaaaggagtttgggggtcaacatggcgtccaggcgctggccagag

L Y - Y S E R S L G V N M A S R R W P E

tggagtgatggcagtaccagtggaggatcttcatacaattgtgaagttactaagaaaggg

W S D G S T S G G S S Y N C E V T K K G

caaacaagtggcaatacgaaccagggacgtcaaaacagaagtcaaggcaatcaagccagc

Q T S G N T N Q G R Q N R S Q G N Q A S

gacaggaagcacaatccatggcaaaatggcaaccaaatggctgttgcaaaaactgctaac

D R K H N P W Q N G N Q M A V A K T A N

tgggttgccactggcacctgggggagcggtcagacgaacgttggaaatgccgctcctccc

W V A T G T W G S G Q T N V G N A A P P

cttatttctggagaagaatgtttctccgagtcaccgtcggagtcttccacagcttggaca

L I S G E E C F S E S P S E S S T A W T

accccaagtggaacaaccagcgtcaatgccagggttgaatctcagcctccaattctatat

T P S G T T S V N A R V E S Q P P I L Y

cagaatgtaaatgtgaaaggcgataatgactttcttgacgacggcagcgaaggaagtgac

Q N V N V K G D N D F L D D G S E G S D

gaagaggcaggtgatcctgtgcacgagcacctctatgctgacttcaagaaatgtgctcaa

E E A G D P V H E H L Y A D F K K C A Q

gactggacagaagaggagatggaggatggccgaatgaactgcccaatctgtaaggcaaaa

D W T E E E M E D G R M N C P I C K A K

ccaggaggcatcaaatggtggcatgggctacgaaaattgattcatcatgcaaaggacttc

P G G I K W W H G L R K L I H H A K D F

cagaagttccgaacaagtgctcatagagagtttgctgagagcattggttatctacttgag

Q K F R T S A H R E F A E S I G Y L L E

aaacggaacatgccactctatggcctttttggcgaagcaccaaaagaaatgtgggttggc

K R N M P L Y G L F G E A P K E M W V G

cctgatgactgggaagaagaaggagatgctcatcgaattttgtggccacctatggtgatt

P D D W E E E G D A H R I L W P P M V I

gtgagaaacacacacttgtaccttgacggagataaatgggttgggatggggaatcctgag

V R N T H L Y L D G D K W V G M G N P E

cttaagaagaagttctctgcattcaagtttgcttcttcttatcacgcttatggacctaag

L K K K F S A F K F A S S Y H A Y G P K

ggccacagggggcttagccttcttgtcttcaagcccagtcatgagggctgggtagaggca

G H R G L S L L V F K P S H E G W V E A

gatgctttggataagatcttctacaaacgcggatttgggagacggcaatggatgactatg

D A L D K I F Y K R G F G R R Q W M T M

ctgcctgacagagttatccgtgatccaaatagaaagaagttcctgtatgcctacctcgca

L P D R V I R D P N R K K F L Y A Y L A

gaagctcgggacattgaagacttcaacaagcatcgtcaagaaaaggacgtgatgaagtat

E A R D I E D F N K H R Q E K D V M K Y

gaacttaagctgtacagcgatgtcattcttcaccctgtgagggcagcagagggagagaga

E L K L Y S D V I L H P V R A A E G E R

gcaagcctcaagtccattcatcaggagagcatgaaggcagtggaggagaacaaactgctg

A S L K S I H Q E S M K A V E E N K L L

cggggacagaaccgtgatatggctcaaaggatcaagctccgggagcaagatcttgagaat

R G Q N R D M A Q R I K L R E Q D L E N

cgcaggaggcagctgaaagctttacaagaaaagcatgctgctgaggaagtgaatgcattg

R R R Q L K A L Q E K H A A E E V N A L

gaggaaattgagagagccaaggaagatgaagcaaaaatctacaacgaaaagcttaatcaa

E E I E R A K E D E A K I Y N E K L N Q

gcttatatgctactggacaaggttcttaacgatgcacatgctaagctggcagacaaaacc

A Y M L L D K V L N D A H A K L A D K T

aaggagctggtggctgcagagagtcagaaggtgccctcagatagcaaacgggaagagttg

K E L V A A E S Q K V P S D S K R E E L

aatcggcaggtcagtgcgctccttctccaagtggaaagctgccaacacgggcagggggag

N R Q V S A L L L Q V E S C Q H G Q G E

tatgtggaacggcttgagggtattctgacggttcagcatgagcgcatgaggatattcaaa

Y V E R L E G I L T V Q H E R M R I F K

gaaaagatgcacaacgaagaacaggaactccatcaaaagaaccgagaggagattgaggca

E K M H N E E Q E L H Q K N R E E I E A

ttgtacaaggaatgcatggctatgatgaagactggatgaggccaacttgccatgatagag

L Y K E C M A M M K T G - G Q L A M I E

gagcagccatcaactagagctgaactgaatcttctagtgaacttaaacatctgaaggaat

E Q P S T R A E L N L L V N L N I - R N

atgggtatgctctgtcagtaaaagagataatgttttttcttccatgttataaagggtcgt

M G M L C Q - K R - C F F F H V I K G R

agatgcatggtttcaaacttgagcttaccctaagtaaatgttactaggaacactcagaag

R C M V S N L S L P - V N V T R N T Q K

gtaacgaacaccctacttttagagggtaataagtcaaactcaatgcttgcatatagaatt

V T N T L L L E G N K S N S M L A Y R I

tccgtgacaaggattgtccagggtatgagcataaatttctctgtagttaaactttatgct

S V T R I V Q G M S I N F S V V K L Y A

cgcatataaaatcttcataacagggactttccagggtatgagcatgaatggctgtgcact

R I - N L H N R D F P G Y E H E W L C T

tg

***Chara vulgaris* (class** [**Charophyceae**](https://www.ncbi.nlm.nih.gov/Taxonomy/Browser/wwwtax.cgi?mode=Undef&id=304574&lvl=3&lin=f&keep=1&srchmode=1&unlock)**)**

**MWXT_2075511**

cttgttagggacgtctgtgaagttgaggtgaatcatcacgagttcctgagttttgcctcaga

C - G R L - S - G E S S R V P E F C L R

gcagagagtgagtgcattatggacgatgcgggccaaatgacattttctataggggacgat

A E S E C I M D D A G Q M T F S I G D D

gtggatgagagtaggttagacagacaagagctcttggctctggaagaaactgcgaggaag

V D E S R L D R Q E L L A L E E T A R K

acgttcgaagagtcgttgcaaaagcaacaagcagaagggaagatatctggagatgtattg

T F E E S L Q K Q Q A E G K I S G D V L

aaggtgcagaagcagcataaatgtgaggcatgcccagcgaagtcgcagagcatatttttg

K V Q K Q H K C E A C P A K S Q S I F L

agtttccaggcgctgataaatcatgcacagtcattcaagaagaagaactggcacattcac

S F Q A L I N H A Q S F K K K N W H I H

agagggtacgcaaaggcgctagaggccttccggaaaaggcacatggaagcaccagaaaat

R G Y A K A L E A F R K R H M E A P E N

gcagtaaaagttgagcaagggcaagatggagaggtggtgtggcctccgacagtcatcttg

A V K V E Q G Q D G E V V W P P T V I L

tgtaatacattcaggagccaagaaggacaagccaaatggtctagcttaggaaaacagcag

C N T F R S Q E G Q A K W S S L G K Q Q

ctgccgccagttctcttgatgctaatggaaggtaaaggaagattccgcccgatctacacg

L P P V L L M L M E G K G R F R P I Y T

aaagcaggtcacaggg

K A G H R
