# Supplementary material for: TAS3 miR390-dependent loci in non-vascular land plants: towards a comprehensive reconstruction of the gene evolutionary history
Source: PeerJ. 2018 Apr 16;6:e4636. doi: 10.7717/peerj.4636 (PMC5907777; doi:10.7717/peerj.4636)
Supplement: Supplemental Information 1 [file peerj-06-4636-s007.doc]

***Sphagnum angustifolium*** Sphan-285 **(accession MF682529)**

GGCGGTAACCCTTCTGAGCTAAGTTTAAACGGATAGGGTTTGTGTTTTGCAAGTAGATTTGTGTGTTTTTTAATGTCTTTTAGTAAGGAAGGAAGCTGAATGTTAGGGTTAACATAATTATTATGTTTTTAGTATAAGCCCTTGTTTCAGATATGAATTCTATAGCTTGAAGACATGACAAACATGTTGTTCGTCATCTCATGATCACCTGCAGACCTACCCTTGAGACAAAATGTTTGCACATTATTGCAACATCTTGTCAATTTAGTTATCACTCCTGAGCTA

***S. girgensohnii*** Sphgi-292 (**accession MF682530)**

GGCGGTAACCCTTCTGAGCGTAAGTTTAAGCAAGATAGGGTTTGTGTTTTGCAAGTAGATTTGTGTATGTGTTTTTTAATGTCTTTCAGAAAGGAAGGAAGCTGAATGTTAGGGTTAACATAATTATTTATGTTTTTAGTATAAGCCCTTGTTTCAGATTTGAATTCTATAGCTTGAAGACATGACAAACATGTTGTTCGTCATCTCATGATCACCTGCAGACCTACCCTTGAGACAAAGTGTTTGCACATTATTGCAACATCTTGTCAATTTAGTTATCACTCCTGAGCTA
